# Supplementary material for: PD-L1 and ICOSL discriminate human Secretory and Helper dendritic cells in cancer, allergy and autoimmunity
Source: Nat Commun. 2022 Apr 13;13:1983. doi: 10.1038/s41467-022-29516-w (PMC9008048; doi:10.1038/s41467-022-29516-w)
Supplement: Supplementary file 1 — Supplementary Information [file 41467_2022_29516_MOESM1_ESM.pdf]

Supplementary Information file

Supplementary Figures 1 to 18	Page 2 - 26
References for Supplementary Figures	Page 27
Supplementary Tables 1 to 10	Page 28 - 37

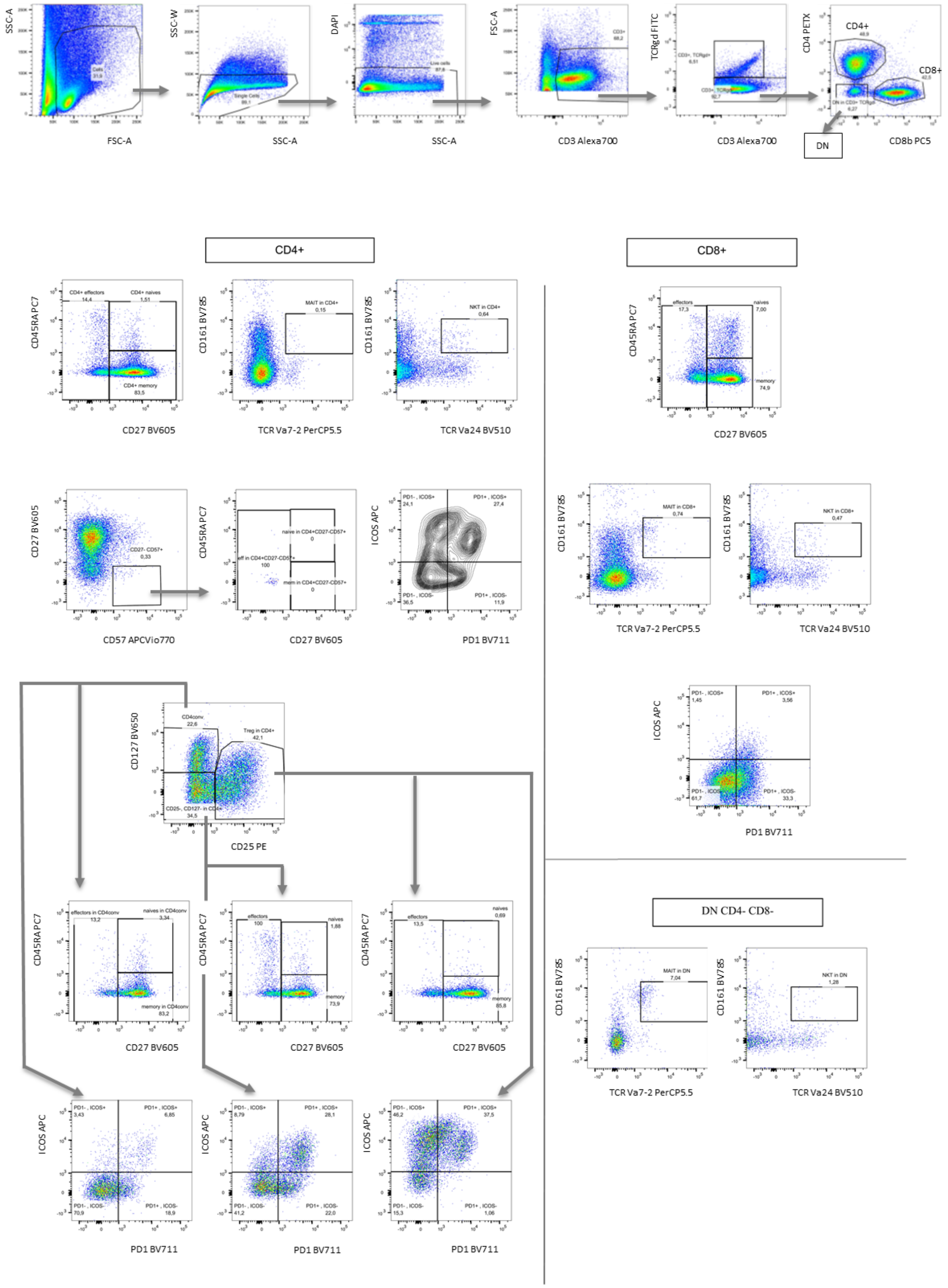

Supplementary Fig. 1. T cell panel gating strategy. Abbreviations: DN = double negative; CD4+ = CD4 T cells; CD8+ = CD8 T cells; NKT = natural killer T cells; eff = effector; mem = memory; conv = conventional; Treg = regulatory T cells.

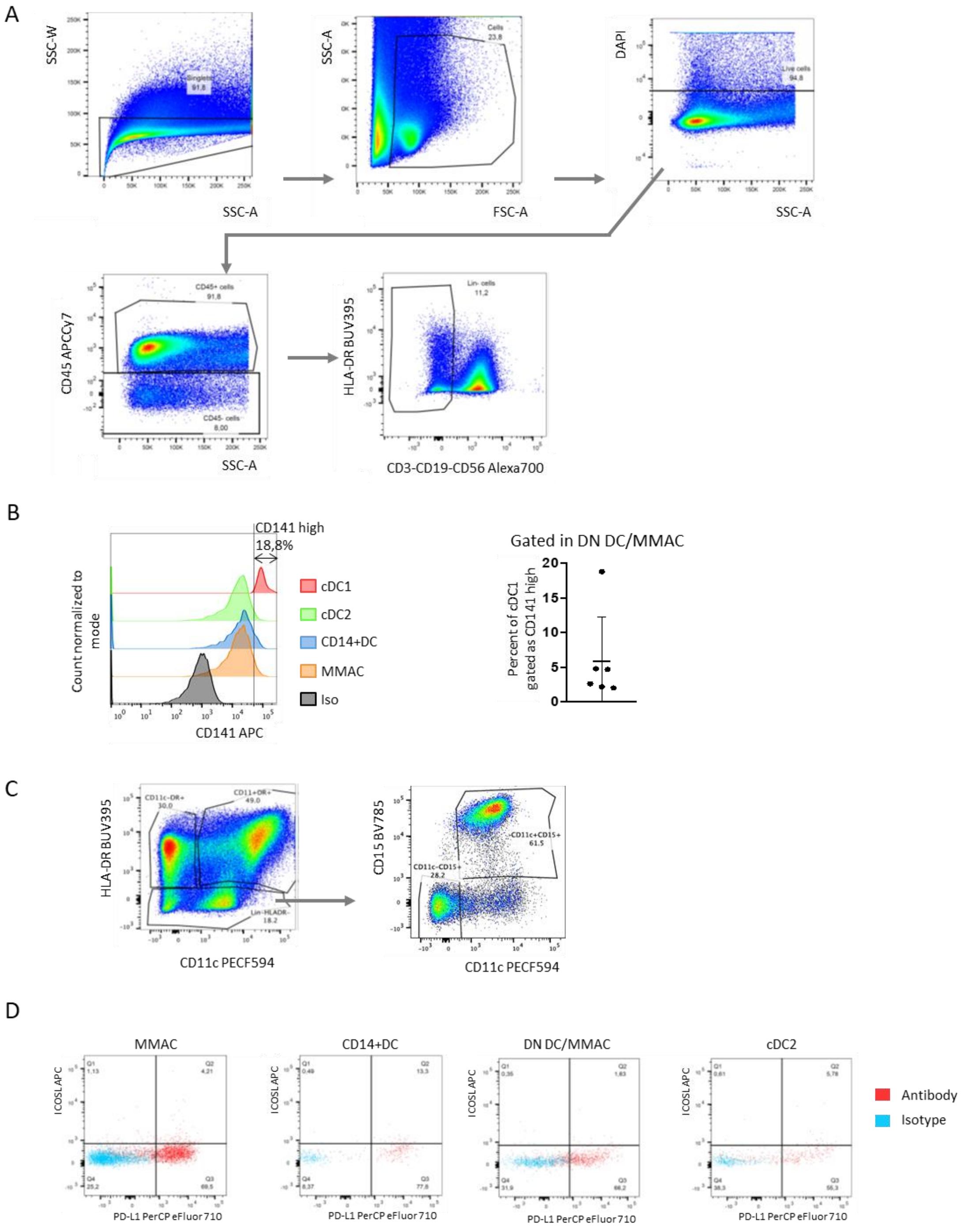

Supplementary Fig. 2. A. Initial gates for Myeloid panel gating strategy before the gates presented in Fig 1B and S2B-D. Lin = lineage and corresponds to CD3, CD19, and CD56. B. Left: Flow cytometry staining for CD141 expression in 4 cell populations in a head and neck squamous cell carcinoma primary tumor. This tumor was selected for its high level of cDC1 infiltration. MMAC = monocytes and macrophages. Right: Percentages of cDC1, gated as CD141 high, in the double negative DC and MMAC (DN DC/MMAC) gate (see Fig 1B) (n = 6 tumors). Central line represents mean, error bars represent standard deviation. C. Flow cytometry staining showing CD15 expression in Lin$^-$HLA-DR$^-$ population. Most Lin$^-$HLA-DR$^-$CD11c$^+$ cells were CD15$^+$, therefore having a neutrophil phenotype. DR = HLA-DR. D. Representative staining of PD-L1 and ICOSL in the four populations gated among CD11c+DR+ cells in a CD3 high tumor: MMAC, CD14+DC, DN DC/MMAC, cDC2 (from left to right), red is for antibody and blue is for isotype control.

A

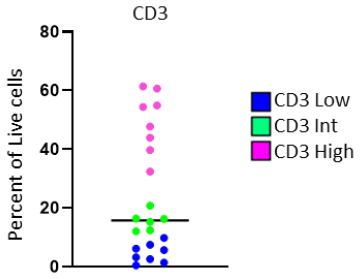

B

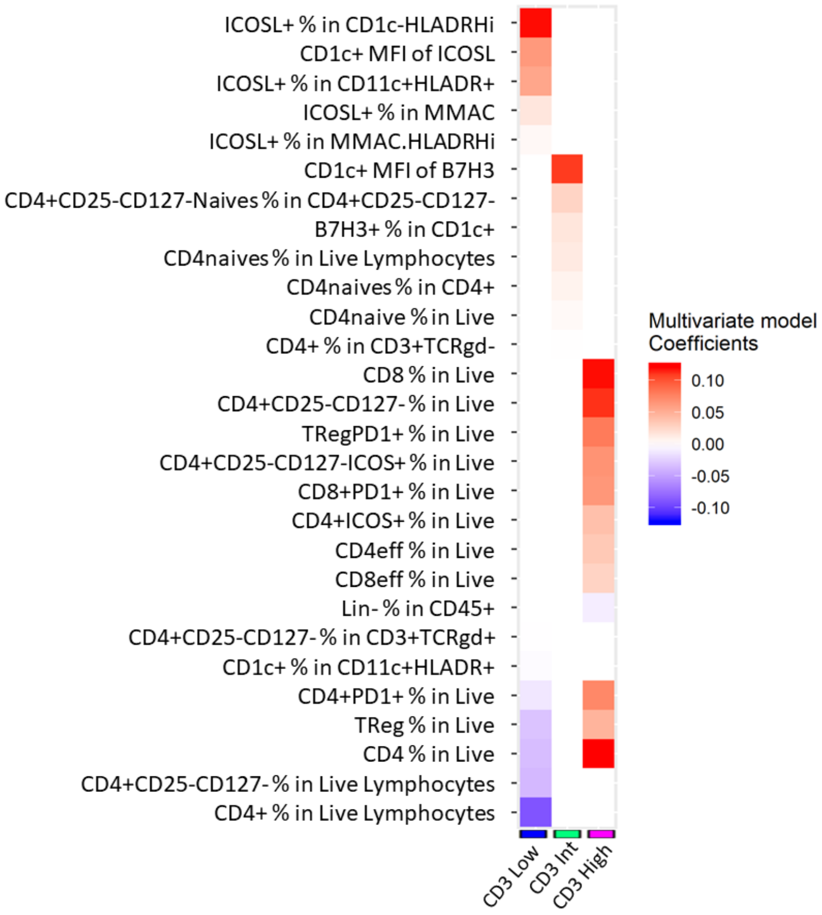

Supplementary Fig. 3. A. Same graph as in Fig 1C representing percentages of CD3+ cells in 22 head and neck squamous cell carcinoma (HNSCC) samples, in which samples were separated by tertile as CD3 high (n=8), int (n=6) and low (n=8). Bar represents median. B. Elastic net model of the 434 parameters measured by flow cytometry and 14 clinical parameters in the 22 HNSCC, showing the parameters the most representative of $CD3^{low}$, $CD3^{int}$ and $CD3^{high}$ tumors. The "Live" gate was established by selecting the live cells among a parental gate of all the cells in the FSC-A versus SCC-A graph, excluding only the debris and red blood cells. The "Live Lymphocyte" gate was established by selecting the live cells among a parental gate of cells having the FSC and SCC levels corresponding to lymphocytes only. Populations names are as defined in Fig S1, S2B and 1B. Abbreviations: Hi = high; MFI = mean fluorescence intensity.

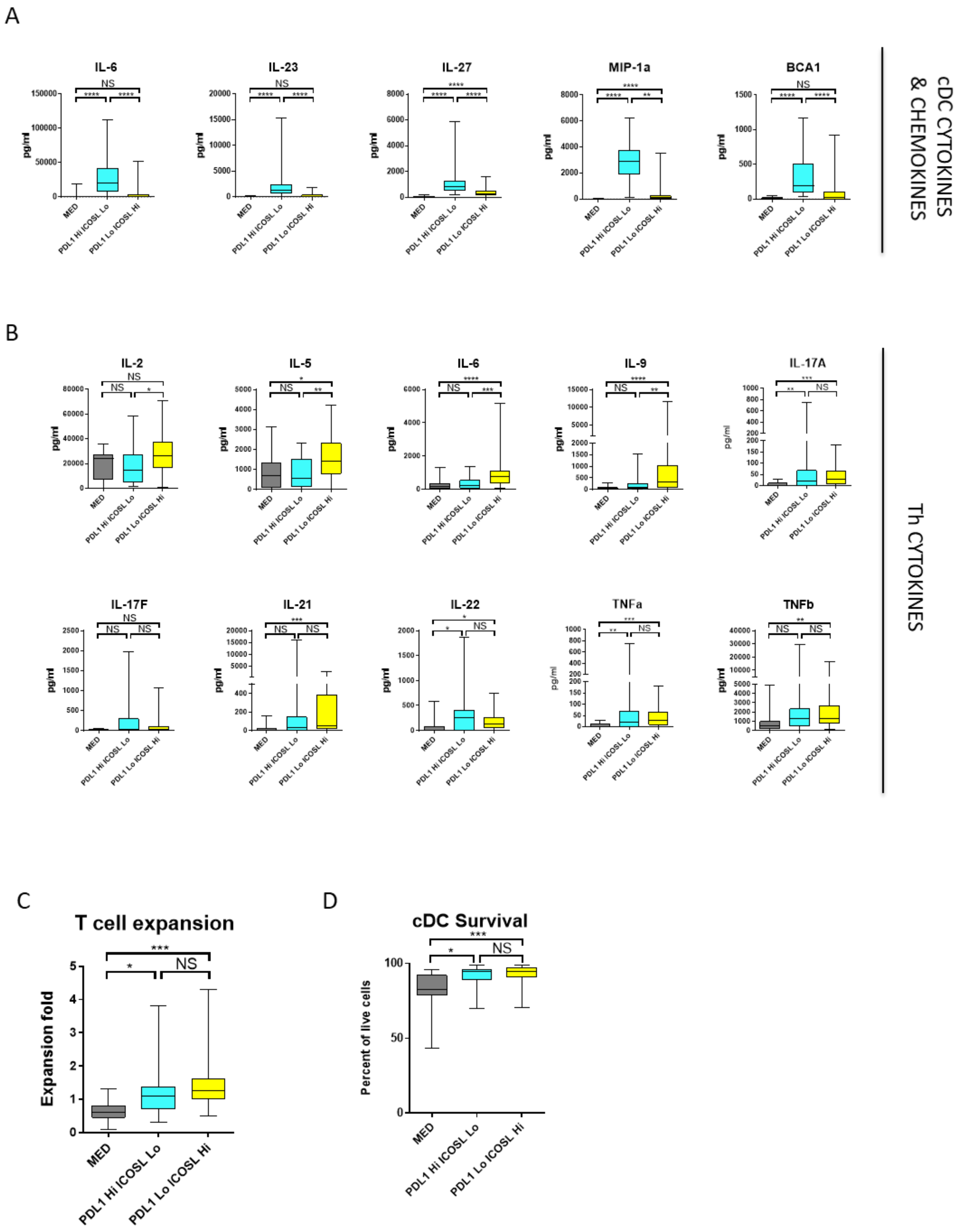
A
IL-6
IL-23
IL-27
MIP-1a
BCA1
pg/ml
MED
PDL1 Hi ICOSL Lo
PDL1 Lo ICOSL Hi
cDC CYTOKINES & CHEMOKINES
B
IL-2
IL-5
IL-6
IL-9
IL-17A
IL-17F
IL-21
IL-22
TNFa
TNFb
Th CYTOKINES
C
T cell expansion
Expansion fold
D
cDC Survival
Percent of live cells

Supplementary Fig. 4. A-D. As in Fig 2B, quantification in the Medium (n=23), PD-L1^{high} ICOSL^{low} (n=38) and PD-L1^{low} ICOSL^{high} (n=40) conditions (two-sided Kruskal-Wallis test on the 3 groups and Dunn's multiple comparison test; central lines represent mean; error bars represent standard deviation). Box represents median and quartiles, whiskers represent min to max. A. Cytokines and chemokines secreted by the cDC. B. CD4 Th cell cytokines. C. T cell expansion at day 6 of DC-T co-culture. D. Percentage of live cDC. p values are represented by range: * < 0.05, ** < 0.01, *** < 0.001, **** < 0.0001. NS = not significant. Abbreviations: MED = Medium; Hi = high; Lo = low.

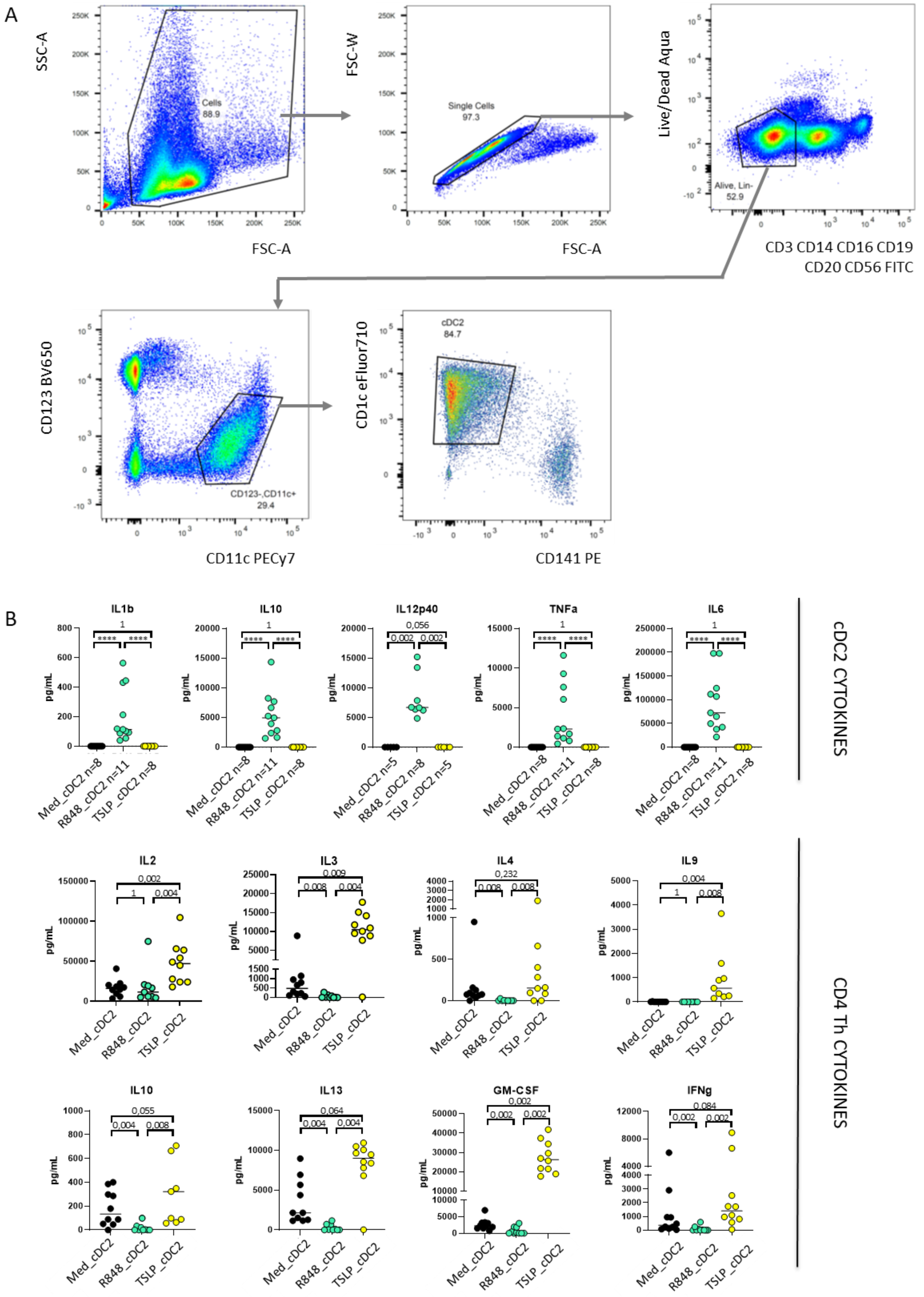
A
SSC-A
FSC-A
Cells
88.9
FSC-W
Single Cells
97.3
FSC-A
Live/Dead Aqua
Alive, Lin-
52.9
CD3 CD14 CD16 CD19
CD20 CD56 FITC
CD123 BV650
CD123-,CD11c+
29.4
CD11c PECy7
CD1c eFluor710
cDC2
84.7
CD141 PE
B
IL1b
IL10
IL12p40
TNFa
IL6
pg/mL
Med_cDC2 n=8
R848_cDC2 n=11
TSLP_cDC2 n=8
Med_cDC2 n=5
R848_cDC2 n=8
TSLP_cDC2 n=5
cDC2 CYTOKINES
IL2
IL3
IL4
IL9
IL10
IL13
GM-CSF
IFNg
Med_cDC2
R848_cDC2
TSLP_cDC2
CD4 Th CYTOKINES

Supplementary Fig. 5. A. Flow cytometry sorting strategy for blood pure cDC2, selected as $CD3^-$, $CD14^-$, $CD16^-$, $CD19^-$, $CD20^-$, $CD56^-$, $CD11c^+$, $CD123^-$, $CD1C^+$, $CD141^-$. Plots from a representative donor. B. Co-culture of pure cDC2 sorted as in Fig S5A with naïve CD4 T cells. cDC2 were treated with R848 (representing Secretory cDC2, "R848_cDC2") or Thymic stromal lymphopoietin (TSLP) (representing Helper cDC2, "TSLP_cDC2"), or untreated ("Med_cDC2"). Top row: Cytokines secreted by the cDC2 at H24, n on graphs, two-sided Mann-Whitney tests. **** is p-value < 0.0001. Two bottom rows: CD4 Th cytokines after 6 days of co-culture and 1 day of restimulation, n = 10 paired samples (except n=9 in the R848_cDC2 condition for IL2, IL3, IL4, IL9 and IL13), two-sided Wilcoxon tests. Bars represent median. Source data are provided as a Source Data file.

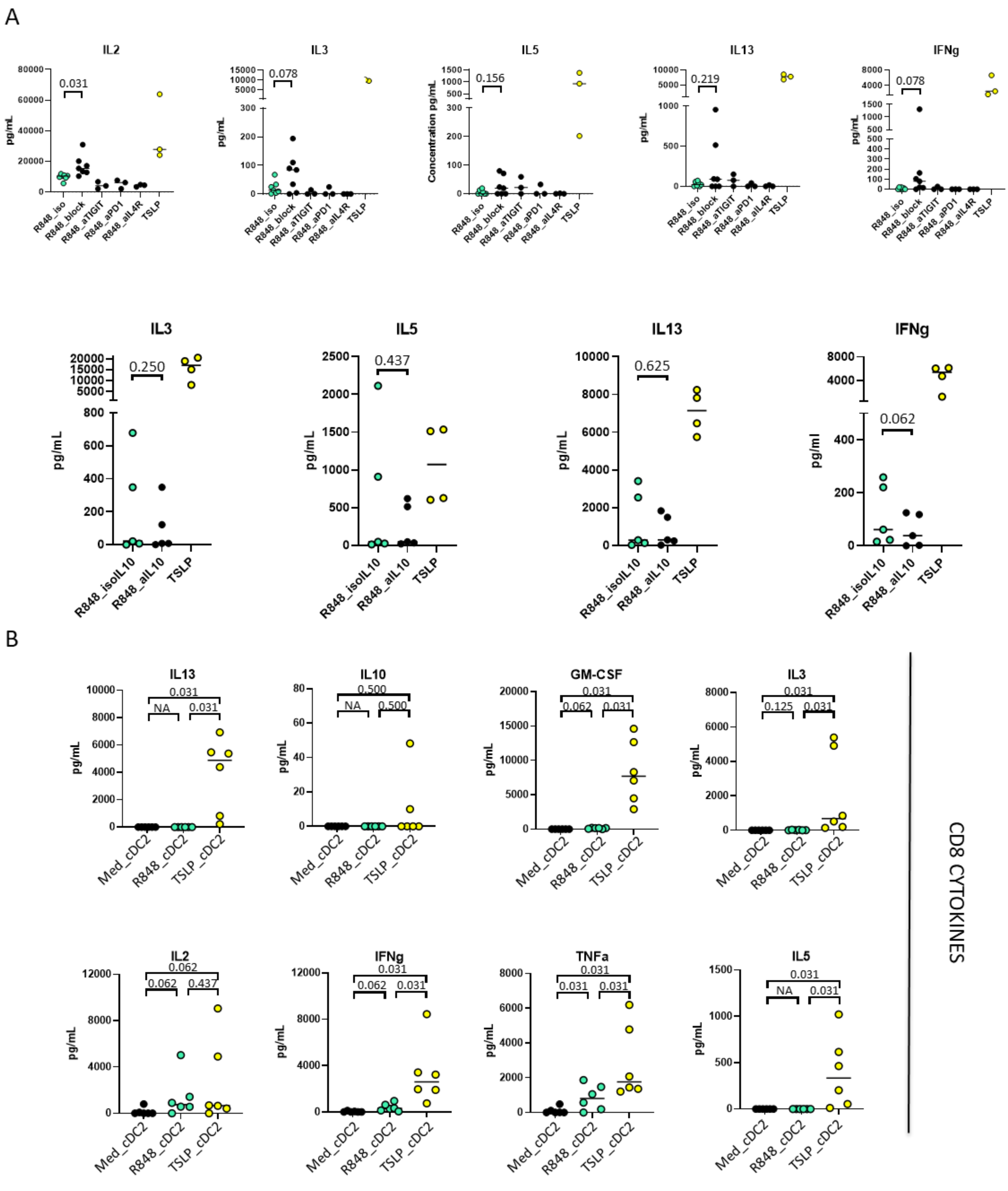

Supplementary Fig. 6. A. Quantification of the CD4 Th cytokines after co-culture with pure cDC2 treated by R848 (representing Secretory cDC2) or Thymic stromal lymphopoietin (TSLP) (Helper cDC2). Top row: TSLP (n=3); for the R848-cDC2, the co-culture was performed in the presence of PD-1; IL-10; IL-10R; IL-4R; TIGIT multiple blockings “R848_block” or the corresponding isotypes “R848_Iso” (n=7, two-sided Wilcoxon test), or single blockings (n=3) of TIGIT (“R848_aTIGIT”), of PD-1 (“R848_aPD1”), of IL-4R (R848_aIL4R). Bottom row: TSLP (n=4); for the R848-cDC2,

the co-culture was performed in the presence of IL-10 and IL-10R blocking (“R848_aIL10”) or the corresponding isotypes (“R848_IsoIL10”) (n=5, two-sided Wilcoxon tests). Bars represent median. B. Quantification of the cytokines secreted by CD8 T cells after co-culture with pure cDC2 treated by R848 (representing Secretory cDC2) or TSLP (Helper cDC2), or untreated (“Med_cDC2”). N = 6, two-sided Wilcoxon tests, bars represent median. Abbreviations: IL-10R = IL-10 receptor; IL-4R = IL-4 receptor; NA: not applicable (all values at zero). Source data are provided as a Source Data file.

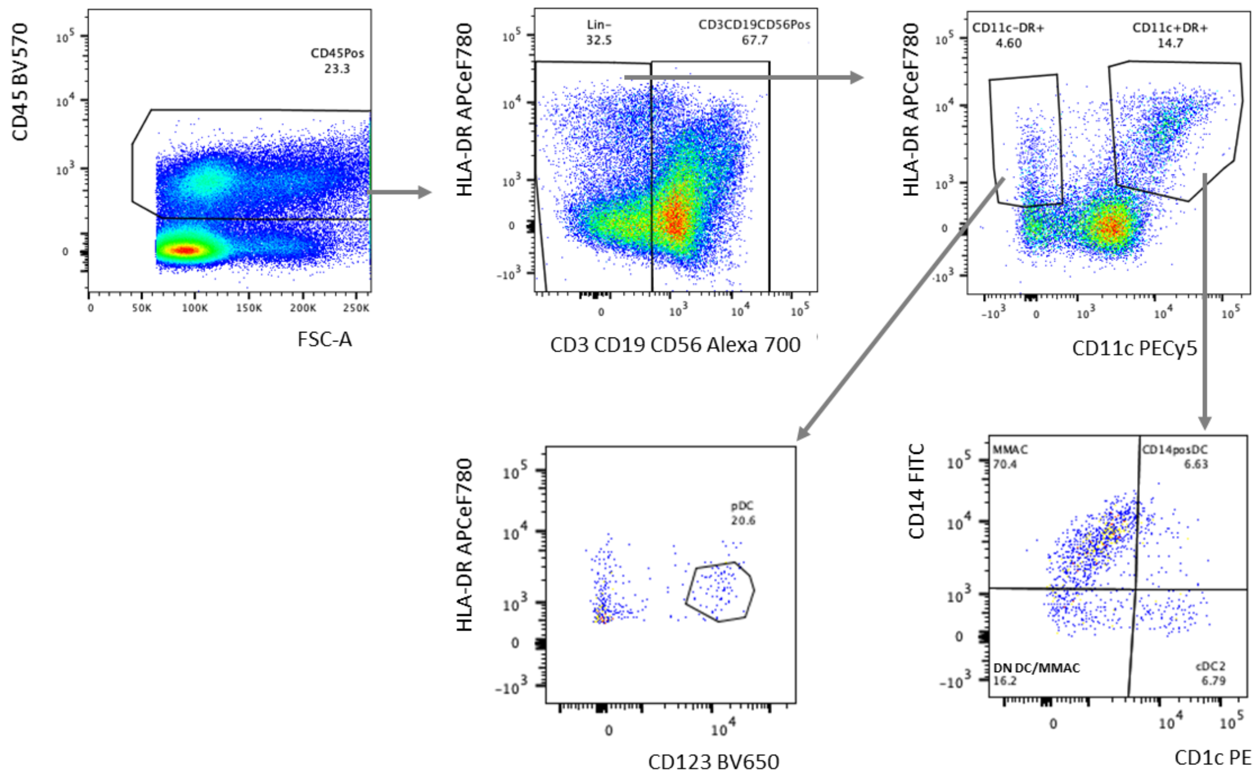

Supplementary Fig. 7. Flow cytometry sorting strategy for RNA sequencing of blood and tumor infiltrating cDC2, selected as CD45^{+}, CD3^{-}, CD19^{-}, CD56^{-}, CD11c^{+}, HLA-DR^{+}, CD14^{-}, CD1C^{+}. Plots from a representative donor. Abbreviations: Pos = positive; DR = HLA-DR; MMAC = monocytes and macrophages; DN DC/MMAC = double negative DC and MMAC.

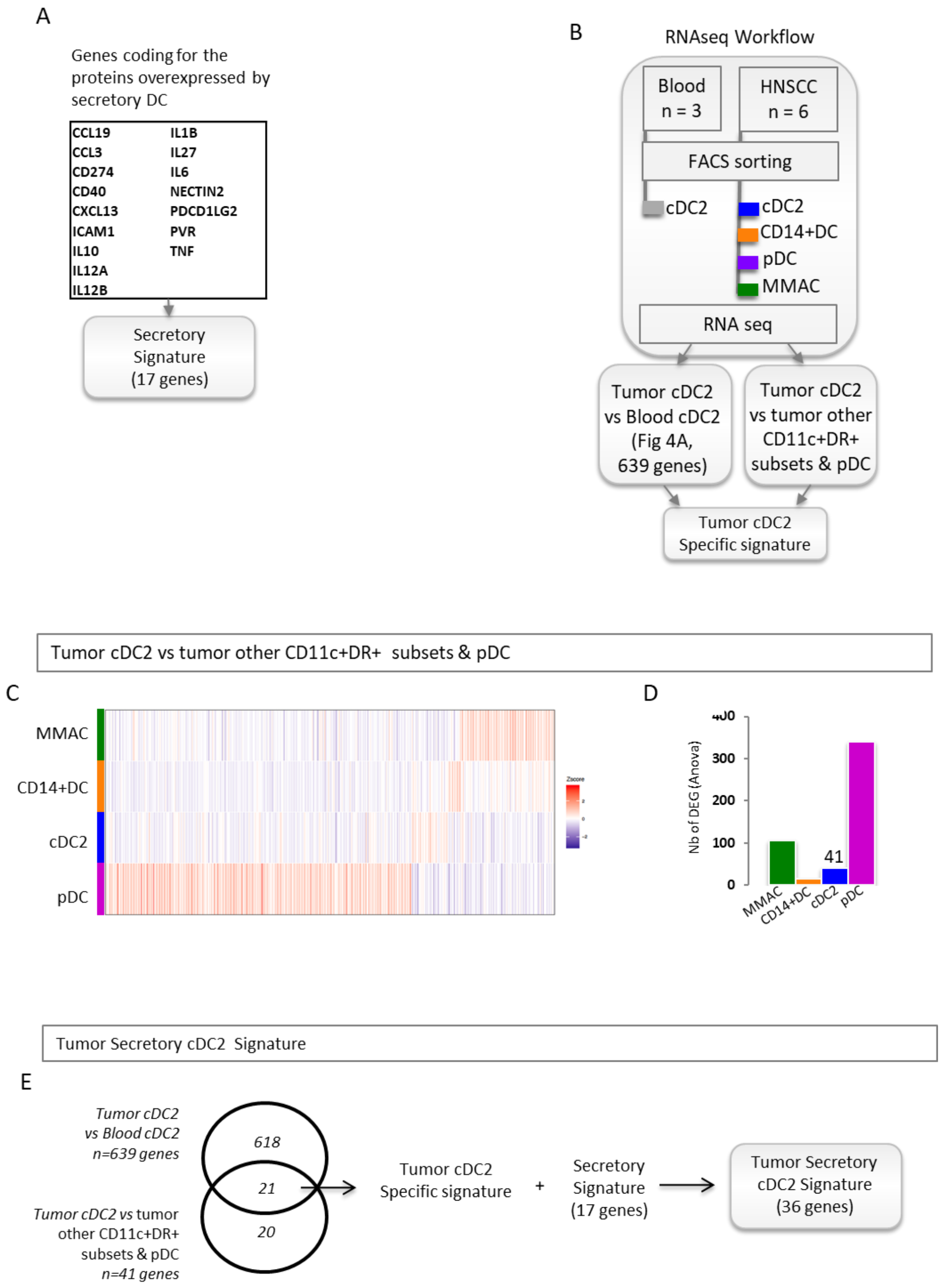

Supplementary Fig. 8. A. List of genes defining the secretory signature. B. RNA sequencing (RNAseq) workflow to define the Tumor cDC2 specific signature from head and neck squamous cell carcinoma (HNSCC) patient samples. C-D. ANOVA between cDC2, other CD11c+DR+ subsets (MMAC and CD14+DC) and pDC: Heatmap (C) and number of differentially expressed genes per subsets obtained by comparing each subset against all others (D). E. Methods to obtain the Tumor Secretory cDC2 signature. Abbreviations: FACS = fluorescence activated cell sorting; pDC = plasmacytoid DC; MMAC = monocytes and macrophages; DR = HLA-DR; DEG = differentially expressed genes.

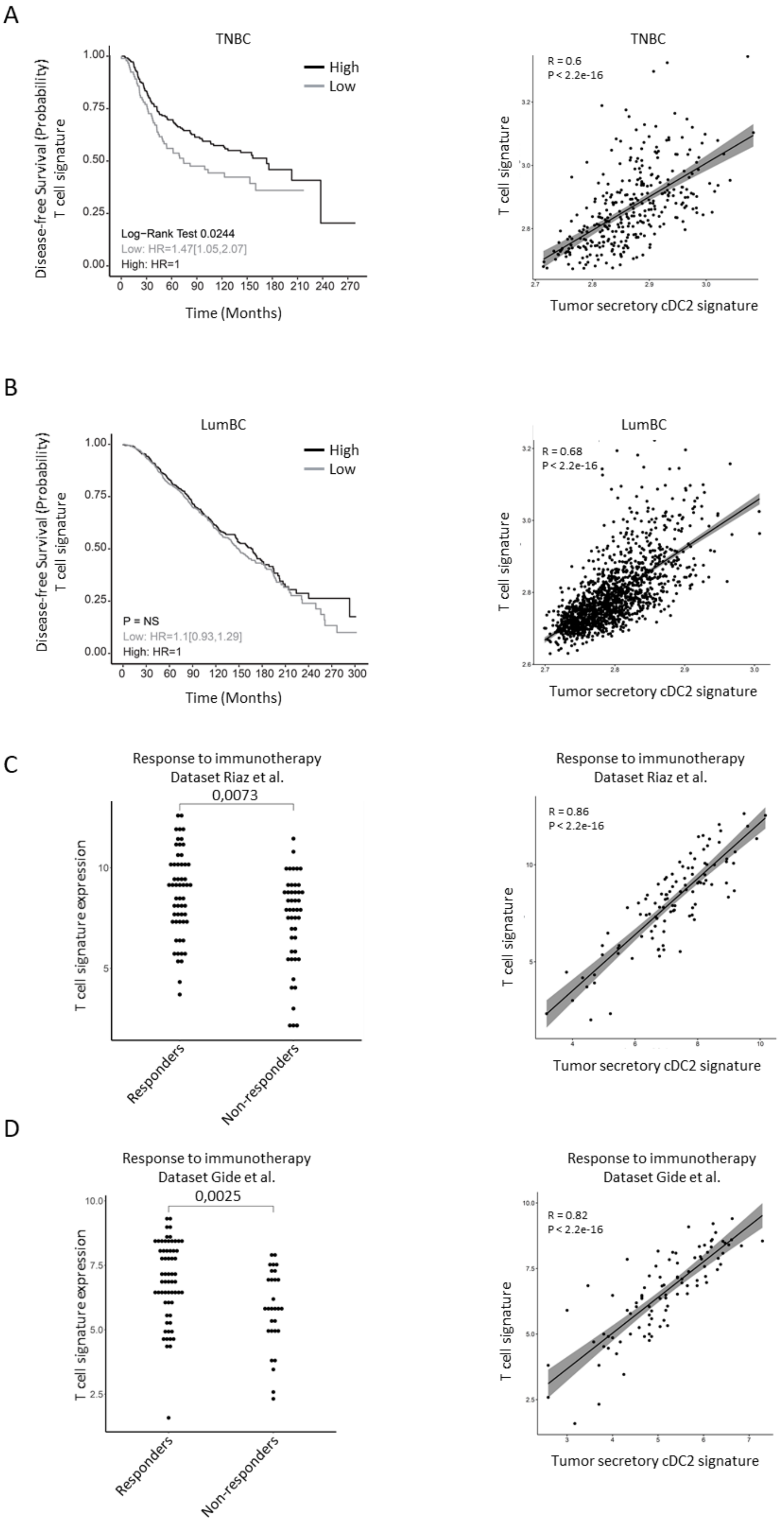
A
TNBC
High
Low
Disease-free Survival (Probability)
T cell signature
Log-Rank Test 0.0244
Low: HR=1.47[1.05,2.07]
High: HR=1
Time (Months)
TNBC
R = 0.6
P < 2.2e-16
T cell signature
Tumor secretory cDC2 signature
B
LumBC
High
Low
Disease-free Survival (Probability)
T cell signature
P = NS
Low: HR=1.1[0.93,1.29]
High: HR=1
Time (Months)
LumBC
R = 0.68
P < 2.2e-16
T cell signature
Tumor secretory cDC2 signature
C
Response to immunotherapy
Dataset Riaz et al.
0,0073
T cell signature expression
Responders
Non-responders
Response to immunotherapy
Dataset Riaz et al.
R = 0.86
P < 2.2e-16
T cell signature
Tumor secretory cDC2 signature
D
Response to immunotherapy
Dataset Gide et al.
0,0025
T cell signature expression
Responders
Non-responders
Response to immunotherapy
Dataset Gide et al.
R = 0.82
P < 2.2e-16
T cell signature
Tumor secretory cDC2 signature

Supplementary Fig. 9. A. Left: Survival analysis among patients expressing high (black) and low (grey) levels of the 5-gene T cell signature (cut-off at median, log-rank test), among 318 triple negative breast cancer (TNBC) patients from METABRIC dataset (1). Right: correlation between the T cell and the 36-gene tumor secretory cDC2 signatures in the same dataset. B. Same as in S9A among 1407 luminal breast cancer (LumBC) patients from METABRIC dataset (1). A-B left: Abbreviations: HR = hazard ratio; NS = not significant. C. Left: T cell signature expression among responders (n=56) and non-responders (n=47) melanoma patients treated by immune checkpoint blockade from study (2). Right: correlation between the T cell and the 36-gene tumor secretory cDC2 signatures in the same dataset. D. Same as in S9C in the study (3), with n=62 responders and n=29 non-responders. C-D left: two-sided Mann-Whitney test, ** are p values < 0.01. A-D right correlations: Pearson correlations (R), lines represent linear regression, grey zones represent 95% confidence interval, p-value are for two-sided statistical analyses.

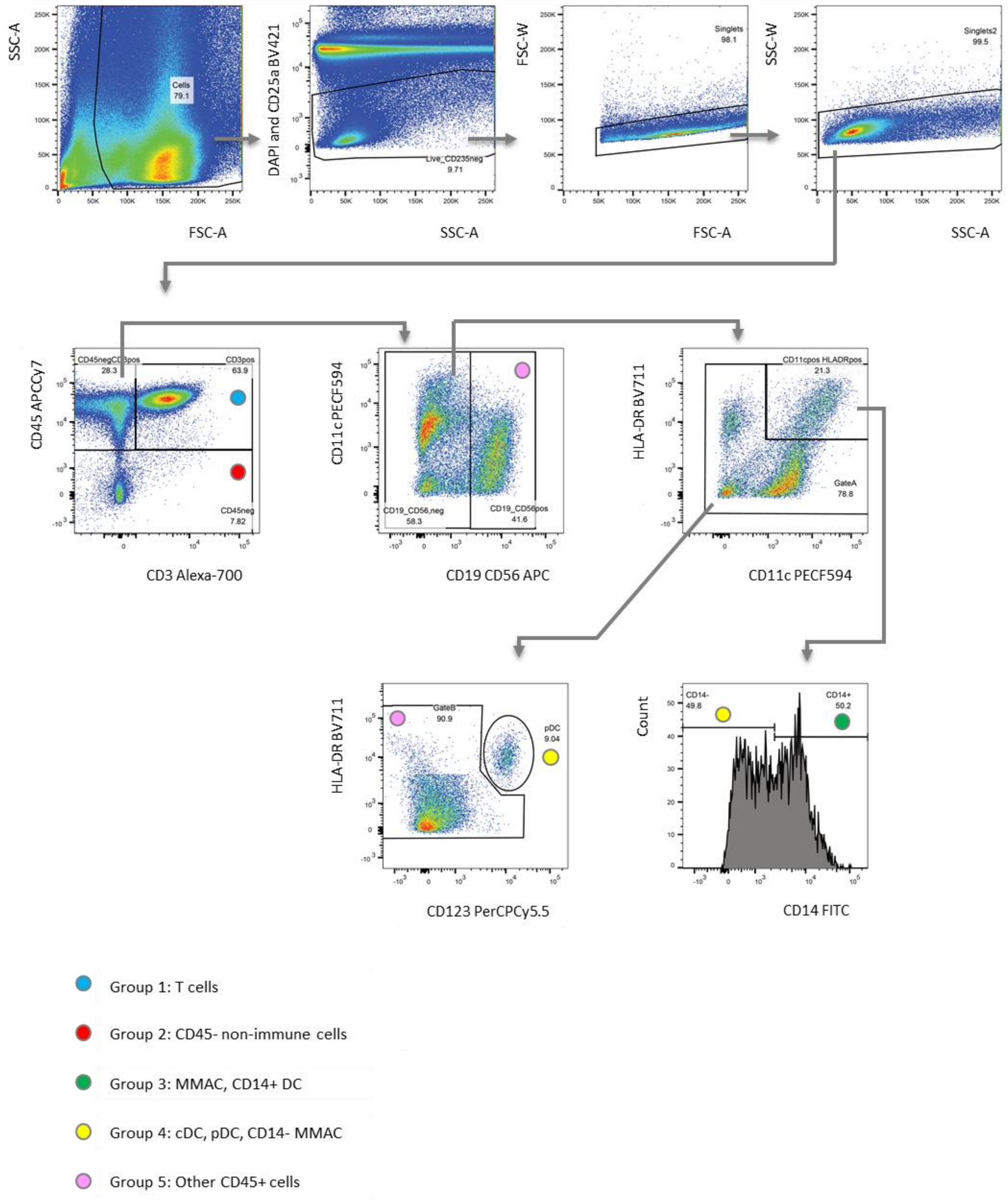

Supplementary Fig. 10. Flow cytometry sorting gating strategy used for DC enrichment before single-cell RNA sequencing. Data from a representative tumor sample. Abbreviations: pDC = plasmacytoid DC; MMAC = monocytes and macrophages.

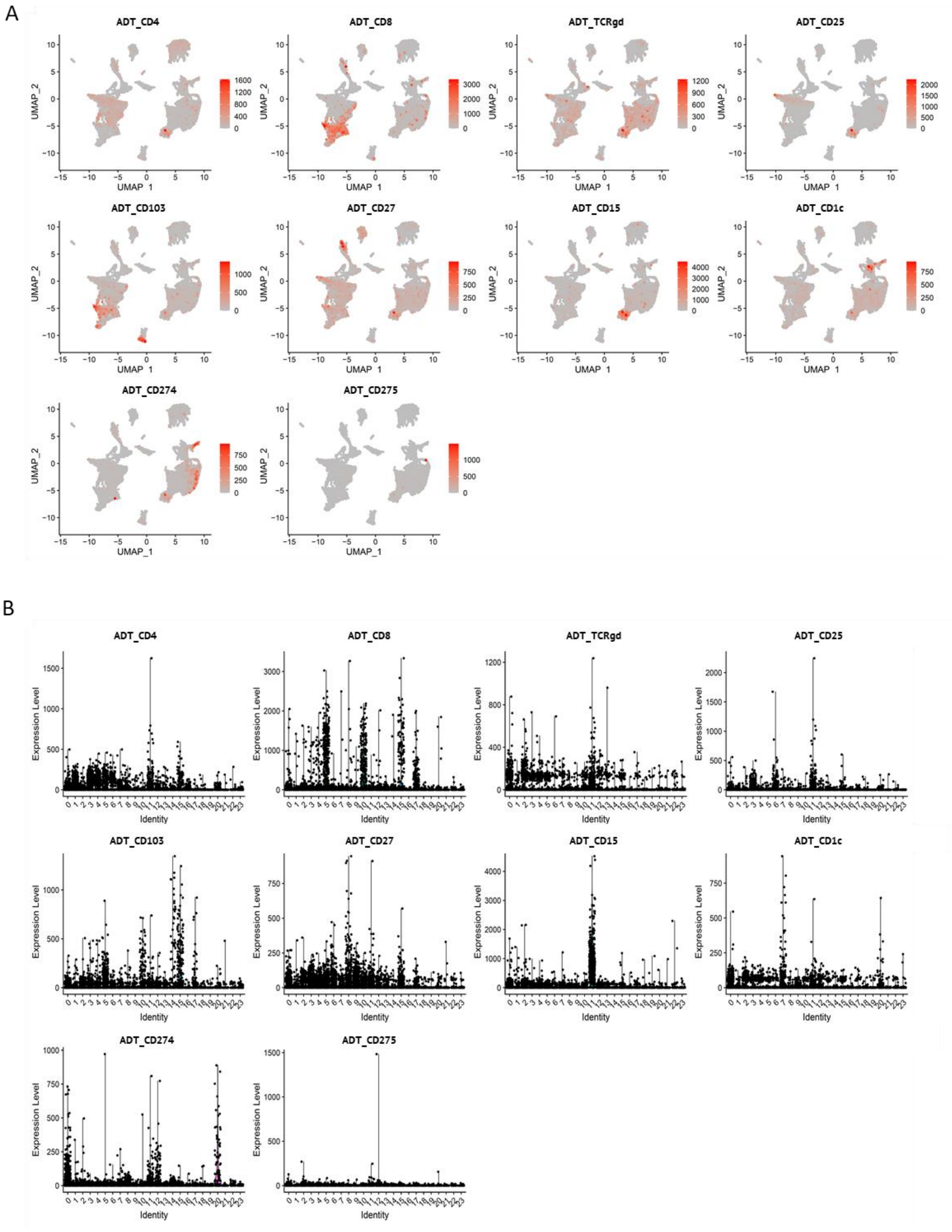

Supplementary Fig. 11. Expression of the 10 antibody-derived tags (ADT) in the single-cell RNA sequencing data, represented A: on the UMAP with all the cells as in Fig 6A; B: in violin plots per cluster.

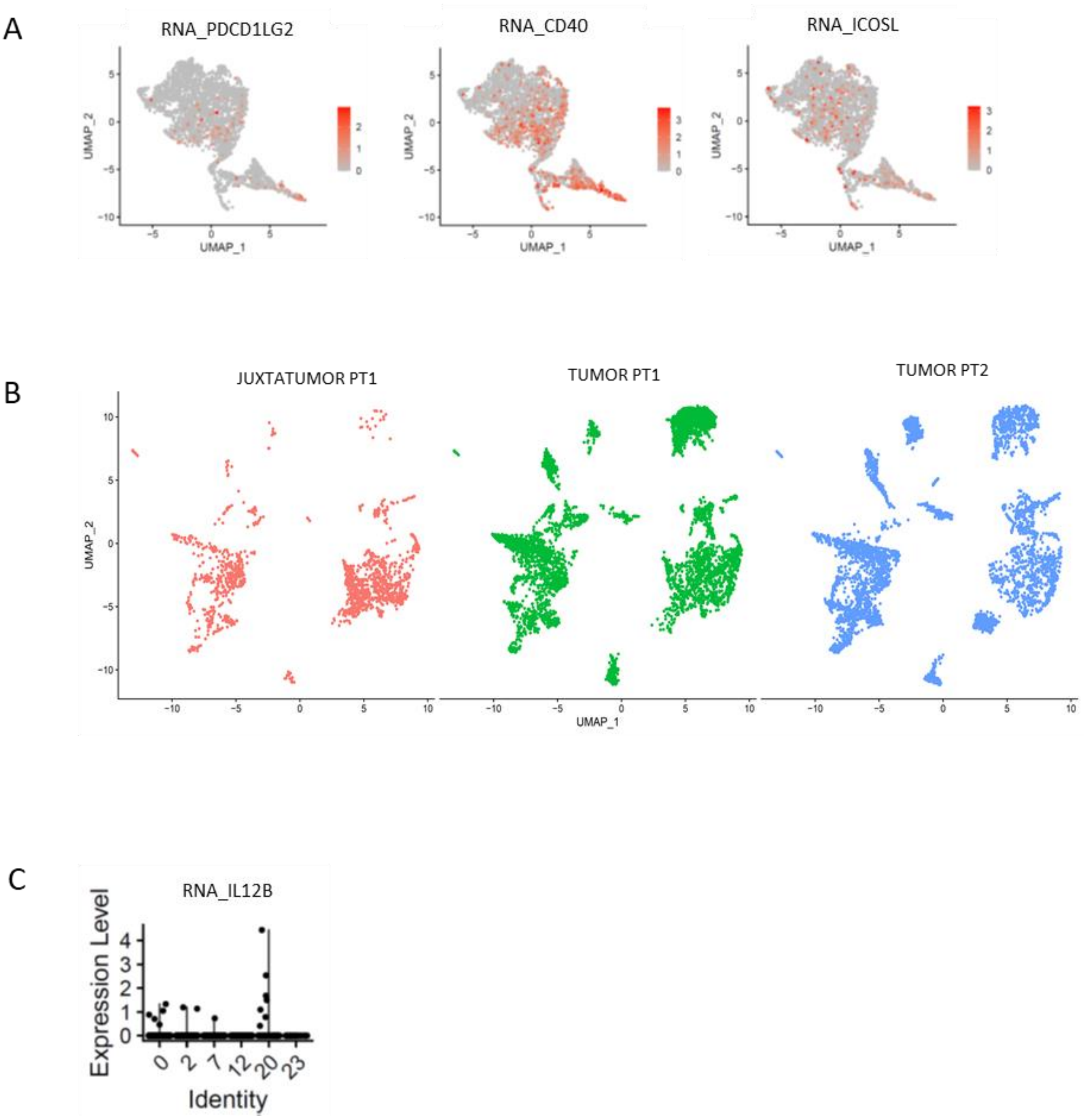

Supplementary Fig. 12. A. Expression of the genes *PDCD1LG2*, *CD40* and *ICOSLG* on the cDC and monocytes and macrophages (cDC/MMAC) UMAP as in Fig 6B. B. Distribution of cells in the UMAP with all cells as in Fig 6A according to the origin of the sample, from left to right: juxtatumor patient 1, tumor patient 1 and tumor patient 2. C. Expression of IL12B in the 6 cDC/MMAC clusters.

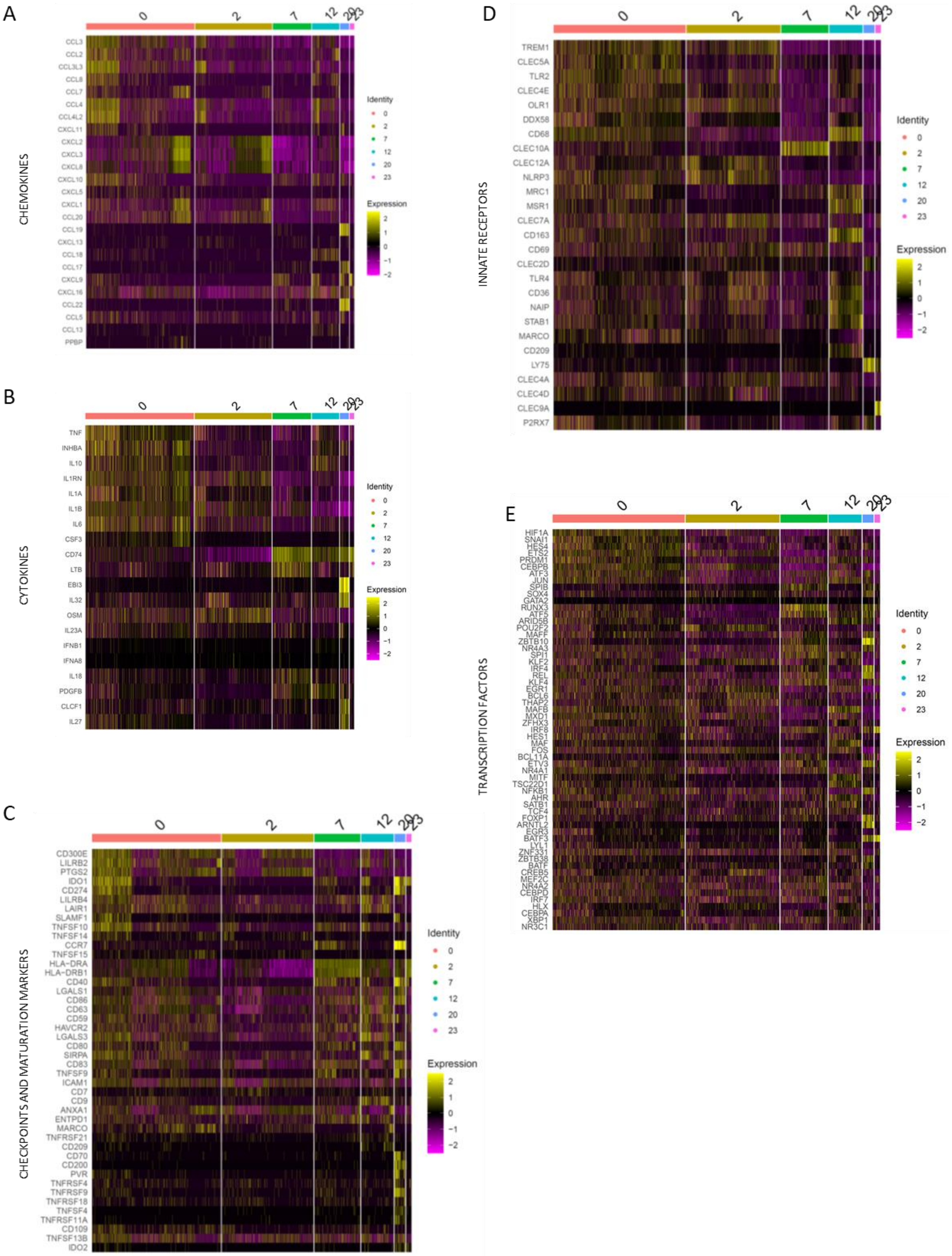
A
CHEMOKINES
CCL3
CCL2
CCL3L3
CCL8
CCL7
CCL4
CCL4L2
CXCL11
CXCL2
CXCL3
CXCL8
CXCL10
CXCL5
CXCL1
CCL20
CCL19
CXCL13
CCL18
CCL17
CXCL9
CXCL16
CCL22
CCL5
CCL13
PPBP
B
CYTOKINES
TNF
INHBA
IL10
IL1RN
IL1A
IL1B
IL6
CSF3
CD74
LTB
EBI3
IL32
OSM
IL23A
IFNB1
IFNA8
IL18
PDGFB
CLCF1
IL27
C
CHECKPOINTS AND MATURATION MARKERS
CD300E
LILRB2
PTGS2
IDO1
CD274
LILRB4
LAIR1
SLAMF1
TNFSF10
TNFSF14
CCR7
TNFSF15
HLA-DRA
HLA-DRB1
CD40
LGALS1
CD86
CD63
CD59
HAVCR2
LGALS3
CD80
SIRPA
CD83
TNFSF9
ICAM1
CD7
CD9
ANXA1
ENTPD1
MARCO
TNFRSF21
CD209
CD70
CD200
PVR
TNFRSF4
TNFRSF9
TNFRSF18
TNFSF4
TNFRSF11A
CD109
TNFSF13B
IDO2
D
INNATE RECEPTORS
TREM1
CLEC5A
TLR2
CLEC4E
OLR1
DDX58
CD68
CLEC10A
CLEC12A
NLRP3
MRC1
MSR1
CLEC7A
CD163
CD69
CLEC2D
TLR4
CD36
NAIP
STAB1
MARCO
CD209
LY75
CLEC4A
CLEC4D
CLEC9A
P2RX7
E
TRANSCRIPTION FACTORS
HIF1A
SNAI1
HES4
ETS2
PRDM1
CEBPB
ATF3
JUN
SPIB
SOX4
GATA2
RUNX3
ATF5
ARID5B
POU2F2
MAFF
ZBTB10
NR4A3
SPI1
KLF2
IRF4
REL
KLF4
EGR1
BCL6
THAP2
MAFB
MXD1
ZFHX3
IRF8
HES1
MAF
FOS
BCL11A
ETV3
NR4A1
MITF
TSC22D1
NFKB1
AHR
SATB1
TCF4
FOXP1
ARNTL2
EGR3
BATF3
LYL1
ZNF331
ZBTB38
BATF
CREB5
MEF2C
NR4A2
CEBPD
IRF7
HLX
CEBPA
XBP1
NR3C1
0
2
7
12
20
23
Identity
0
2
7
12
20
23
Expression
2
1
0
-1
-2

Supplementary Fig. 13. Heatmap of expression of differentially expressed genes from supervised gene lists in the 6 cDC and monocytes and macrophages clusters from Fig 6B. Full gene lists are available in the Supplementary Data mentioned in brackets. A. Chemokines (Supplementary Data 6). B. Cytokines (Supplementary Data 5). C. Checkpoints and maturation markers (Supplementary Data 4). D. Innate Receptors (Supplementary Data 12). E. Transcription factors (1639 genes from (4)).

A

Cillo et al.

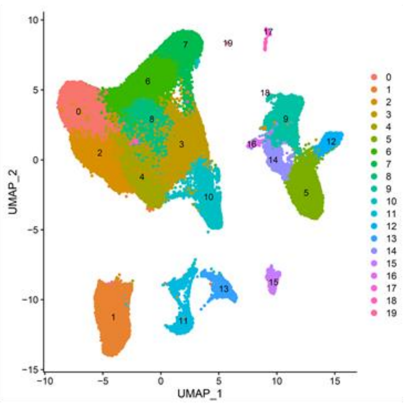

B

Luminal Breast Cancer

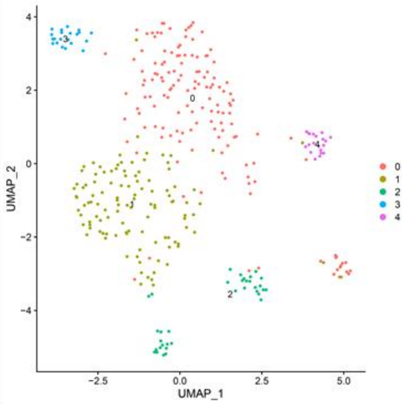

C

He et al.

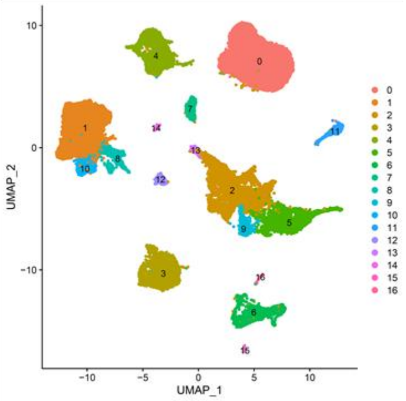

Supplementary Fig. 14. A. UMAP of 113326 cells analyzed by single-cell RNA sequencing (ScRNAseq) from the public dataset from Cillo et al. (5) displayed in 20 clusters. DC clusters 14 (1478 cells) and 16 (394 cells) were selected for downstream analyses. B. UMAP of the 333 dendritic cells analyzed by ScRNAseq of a luminal breast cancer sample displayed in 5 clusters. Cluster 4 corresponded to pDC (20 cells) and has been excluded from downstream analyses. C. UMAP of 39600 cells analyzed by ScRNAseq from the public dataset from He et al. (6) displayed in 17 clusters. DC cluster 6 (1827 cells) was selected for downstream analyses.

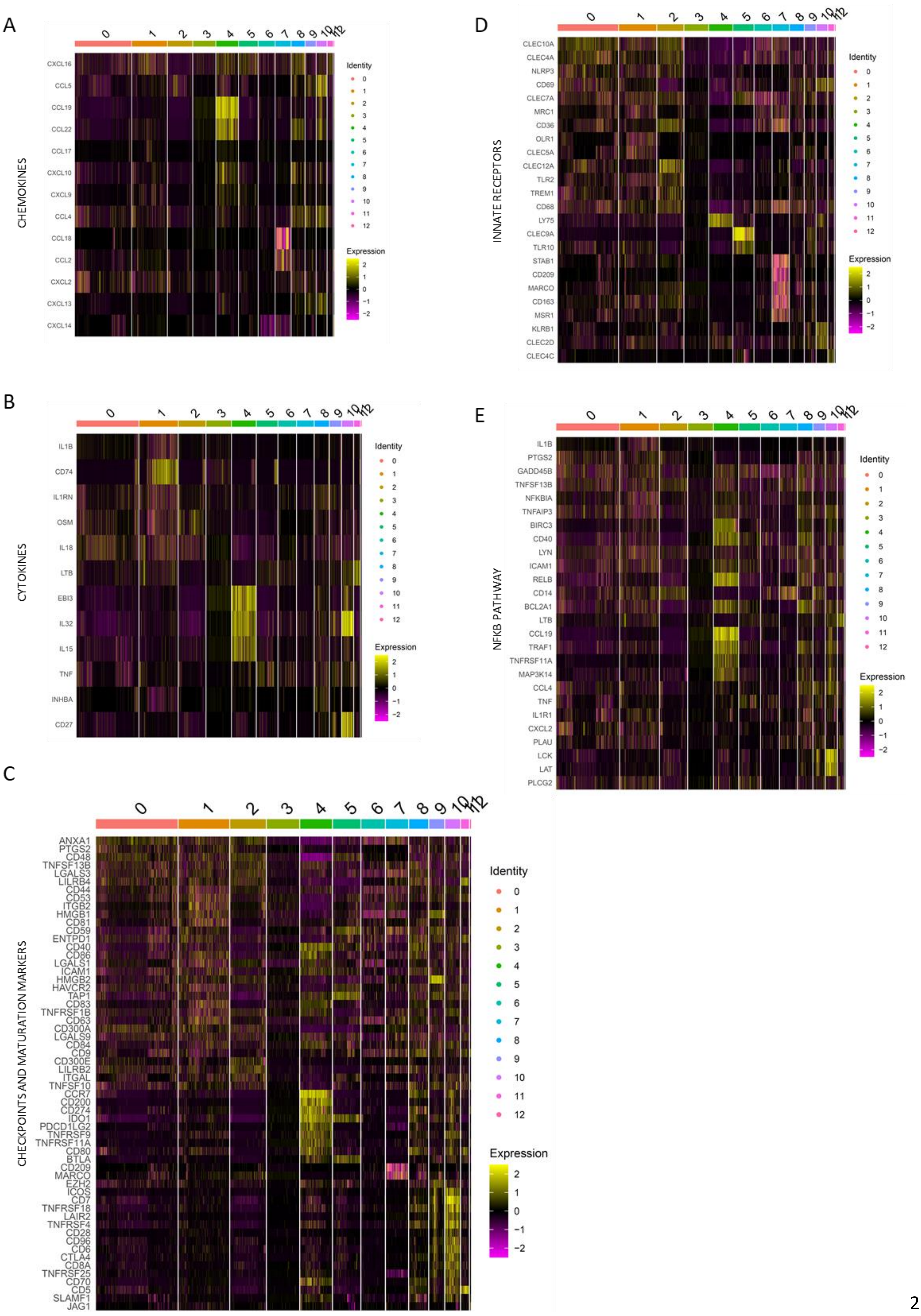
A
CHEMOKINES
CXCL16
CCL5
CCL19
CCL22
CCL17
CXCL10
CXCL9
CCL4
CCL18
CCL2
CXCL2
CXCL13
CXCL14
B
CYTOKINES
IL1B
CD74
IL1RN
OSM
IL18
LTB
EBI3
IL32
IL15
TNF
INHBA
CD27
C
CHECKPOINTS AND MATURATION MARKERS
ANXA1
PTGS2
CD48
TNFSF13B
LGALS3
LILRB4
CD44
CD53
ITGB2
HMGB1
CD81
CD59
ENTPD1
CD40
CD86
LGALS1
ICAM1
HMGB2
HAVCR2
TAP1
CD83
TNFRSF1B
CD63
CD300A
LGALS9
CD84
CD9
CD300E
LILRB2
ITGAL
TNFSF10
CCR7
CD200
CD274
IDO1
PDCD1LG2
TNFRSF9
TNFRSF11A
CD80
BTLA
CD209
MARCO
EZH2
ICOS
CD7
TNFRSF18
LAIR2
TNFRSF4
CD28
CD96
CD6
CTLA4
CD8A
TNFRSF25
CD70
CD5
SLAMF1
JAG1
D
INNATE RECEPTORS
CLEC10A
CLEC4A
NLRP3
CD69
CLEC7A
MRC1
CD36
OLR1
CLEC5A
CLEC12A
TLR2
TREM1
CD68
LY75
CLEC9A
TLR10
STAB1
CD209
MARCO
CD163
MSR1
KLRB1
CLEC2D
CLEC4C
E
NFKB PATHWAY
IL1B
PTGS2
GADD45B
TNFSF13B
NFKBIA
TNFAIP3
BIRC3
CD40
LYN
ICAM1
RELB
CD14
BCL2A1
LTB
CCL19
TRAF1
TNFRSF11A
MAP3K14
CCL4
TNF
IL1R1
CXCL2
PLAU
LCK
LAT
PLCG2
Identity
0
1
2
3
4
5
6
7
8
9
10
11
12
Expression
2
1
0
−1
−2

Supplementary Fig. 15. Heatmap of expression of differentially expressed genes from supervised gene lists in the 13 clusters of the merged DC dataset from Fig 8. Full gene lists are available in the Supplementary Data mentioned in brackets. A. Chemokines (Supplementary Data 6). B. Cytokines (Supplementary Data 5). C. Checkpoints and maturation markers (Supplementary Data 4). D. Innate Receptors (Supplementary Data 12). E. NFkB pathway (Supplementary Data 7).

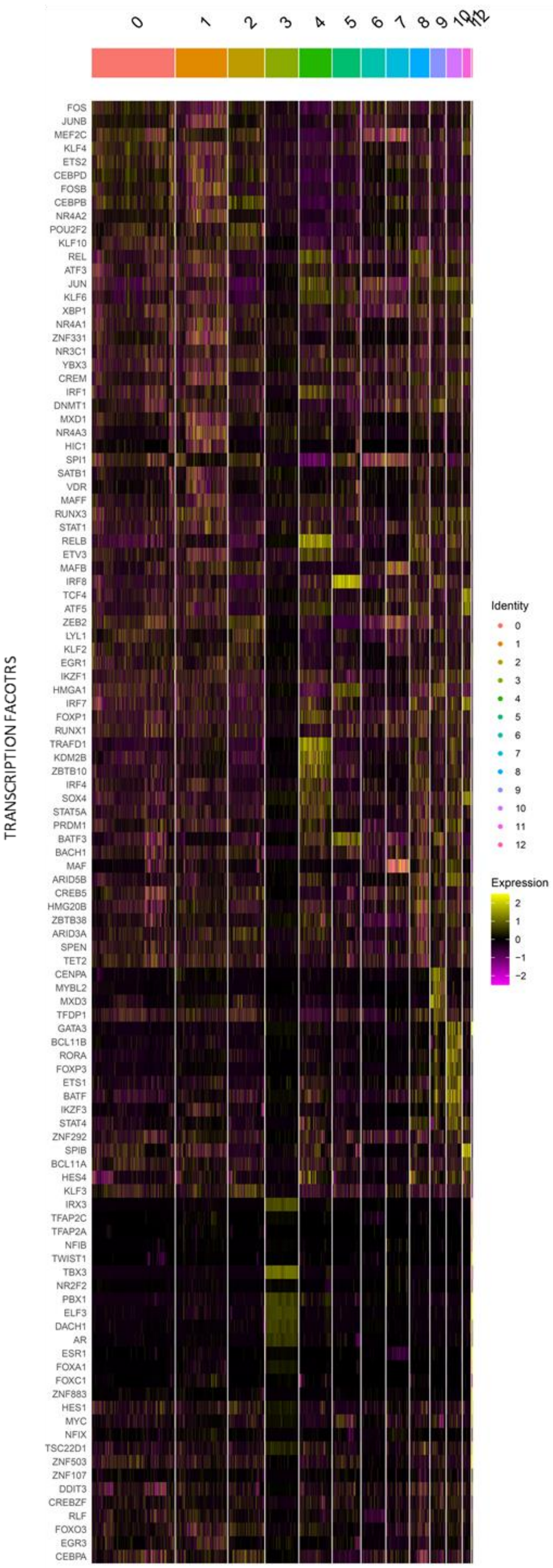

Supplementary Fig. 16. Heatmap of expression of differentially expressed genes of human transcription factors (1639 genes from (4)) in the 13 clusters of the merged DC dataset from Fig 8.

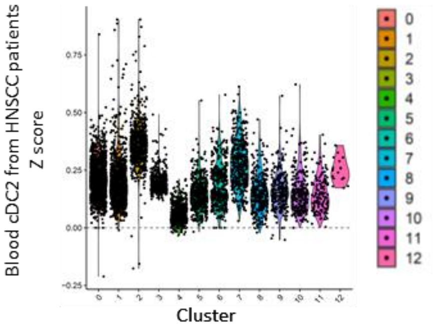

Supplementary Fig. 17. Expression of the Blood cDC2 signature from Fig. 4A in the 13 clusters of the merged DC dataset from Fig 8.

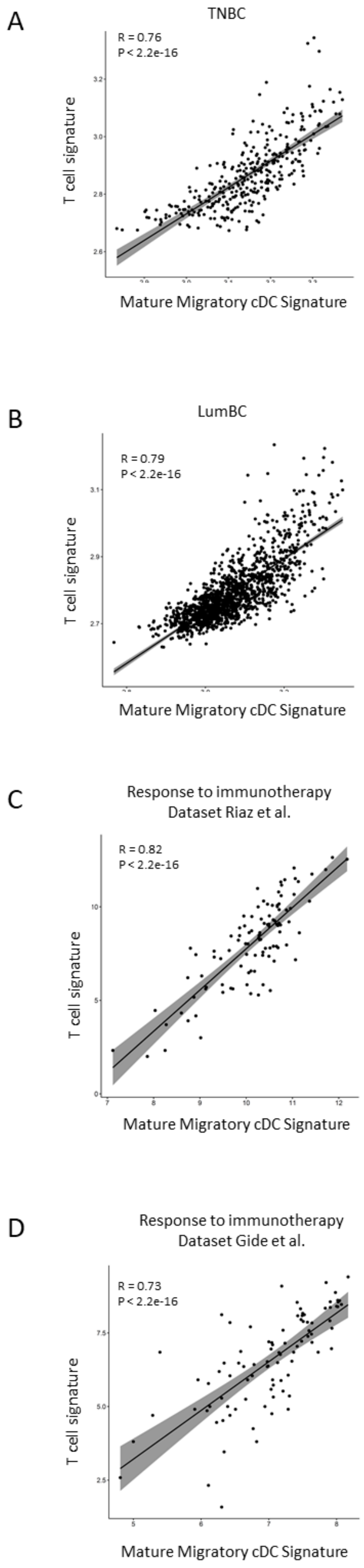

Supplementary Fig. 18. A-D. Pearson correlation (R) between the T cell and the Mature Migratory cDC (Table 10) signatures, lines represent linear regression; grey zones represent 95% confidence interval, p-value are for two-sided

statistical analyses. A. Among 318 triple negative breast cancer (TNBC) patients from METABRIC dataset (1). B. Among 1407 luminal breast cancer (LumBC) patients from METABRIC dataset (1). C. Among 103 melanoma patients treated by immune checkpoint blockade from study (2). D. Among 91 melanoma patients treated by immune checkpoint blockade from study (3).

References for Supplementary Figures

1. Curtis C, Shah SP, Chin S-F, Turashvili G, Rueda OM, Dunning MJ, et al. The genomic and transcriptomic architecture of 2,000 breast tumours reveals novel subgroups. Nature. 2012 Apr 18;486(7403):346–52.

2. Riaz N, Havel JJ, Makarov V, Desrichard A, Urba WJ, Sims JS, et al. Tumor and Microenvironment Evolution during Immunotherapy with Nivolumab. Cell. 2017 Nov 2;171(4):934-949.e16.

3. Gide TN, Quek C, Menzies AM, Tasker AT, Shang P, Holst J, et al. Distinct Immune Cell Populations Define Response to Anti-PD-1 Monotherapy and Anti-PD-1/Anti-CTLA-4 Combined Therapy. Cancer Cell. 2019 11;35(2):238-255.e6.

4. Lambert SA, Jolma A, Campitelli LF, Das PK, Yin Y, Albu M, et al. The Human Transcription Factors. Cell. 2018 08;172(4):650–65.

5. Cillo AR, Kürten CHL, Tabib T, Qi Z, Onkar S, Wang T, et al. Immune Landscape of Viral- and Carcinogen-Driven Head and Neck Cancer. Immunity. 2020 14;52(1):183-199.e9.

6. He H, Suryawanshi H, Morozov P, Gay-Mimbrera J, Del Duca E, Kim HJ, et al. Single-cell transcriptome analysis of human skin identifies novel fibroblast subpopulation and enrichment of immune subsets in atopic dermatitis. J Allergy Clin Immunol. 2020 Jun;145(6):1615–28.

Supplementary Table 1. Cell populations analyzed by flow cytometry

Cell populations	Markers	Checkpoint / Maturation marker	Short name
T cells Panel			
CD3	CD3+		CD3 T
CD3 TCRgd	CD3+TCRgd		Gd T
CD4+	CD3+TCRgd-CD4+CD8-		CD4+ T
CD4 mem	CD3+TCRgd-CD4+CD8-CD27+CD45RA-		CD4+ mem T
CD4 naive	CD3+TCRgd-CD4+CD8-CD27+CD45RA+		CD4+ naive T
CD4 eff	CD3+TCRgd-CD4+CD8-CD27-CD45RA+		CD4+ eff T
T Reg	CD3+TCRgd-CD4+CD8-CD25+CD127int/-	PD1, ICOS	Treg
CD4conv	CD3+TCRgd-CD4+CD8-CD25-CD127+	PD1, ICOS	CD4+CD25-CD127+T
CD25NegCD127Neg	CD3+TCRgd-CD4+CD8-CD25-CD127-	PD1, ICOS	CD4+CD25-CD127- T
CD27NegCD57Pos	CD3+TCRgd-CD4+CD8-CD27-CD57+		CD4+CD27-CD57+ T
MAIT CD4	CD3+TCRgd-CD4+CD8-CD161+TCRVa72+		CD4+ MAIT
NKT CD4	CD3+TCRgd-CD4+CD8-CD161+TCRVa24-		CD4+ NKT
CD4CD161PosTCRVa24Neg	CD3+TCRgd-CD4+CD8-CD161+TCRVa24-		CD4+ CD161+ T
CD8+	CD3+TCRgd-CD8+CD4-	PD1, ICOS	CD8+ T
CD8 mem	CD3+TCRgd-CD8+CD4-CD27+CD45RA-		CD8+ mem T
CD8 naive	CD3+TCRgd-CD8+CD4-CD27+CD45RA+		CD8+ naive T
CD8 eff	CD3+TCRgd-CD8+CD4-CD27-CD45RA+		CD8+ eff T
MAIT CD8	CD3+TCRgd-CD8+CD4-CD161+TCRVa72+		CD8+ MAIT
NKT CD8	CD3+TCRgd-CD8+CD4-CD161+TCRVa24-		CD8+ NKT
CD8CD161PosTCRVa24Neg	CD3+TCRgd-CD8+CD4-CD161+TCRVa24-		CD8+ CD161+ T
DN	CD3+TCRgd-CD4-CD8-		DN T
MAIT	CD3+TCRgd-CD4-CD8-CD161+TCRVa72+		DN MAIT
NKT	CD3+TCRgd-CD4-CD8-CD161+TCRVa24-		DN NKT
Myeloid cells Panel			
CD45Pos	CD45+	B7H3, PDL1	CD45+
LinNeg	CD45+Lin-		Lin-
CD11cPosHLADRPos	CD45+Lin-CD11c+HLADR+		CD11c+DR+
CD1cPos	CD45+Lin-CD11c+HLADR+CD14-CD1c+	B7H3, PDL1, ICOSL, CD86, CD83	cDC2
CD1cPosHLADRLo_CD1cPos	CD45+Lin-CD11c+HLADR+but low CD14-CD1c+		cDC2 DRlow
CD1cNeg	CD45+Lin-CD11c+HLADR+CD14-CD1c-	B7H3, PDL1, ICOSL, CD86, CD83	DN DC/MMAC
CD1cNeg HLADRLo_CD1cNeg	CD45+Lin-CD11c+HLADR+but low CD14-CD1c-		DN DC/MMAC DRlow
CD14PosDC	CD45+Lin-CD11c+HLADR+CD14+CD1c+	B7H3, PDL1, ICOSL, CD86, CD83	CD14+DC
CD14PosDC HLADRLo_CD14Pos	CD45+Lin-CD11c+HLADR+but low CD14-CD1c+		CD14+DC DRlow
MMAC	CD45+Lin-CD11c+HLADR+CD14+CD1c-	B7H3, PDL1, ICOSL, CD86, CD83	MMAC
MMAC HLADRLo_MMAC	CD45+Lin-CD11c+HLADR+but low CD14+CD1c-		MMAC DRlow
pDC_LinNeg	CD45+Lin-CD11c-HLADR+CD123+	B7H3, PDL1, ICOSL, CD86, CD83	pDC
LinNegHLADRNeg	CD45+Lin-HLADR-		Lin-DR-
CD11cPosHLADRNeg	CD45+Lin-HLADR-CD11c+	B7H3, PDL1, ICOSL, CD86, CD83	Neutrophils_e
CD11cNegHLADRNeg	CD45+Lin-HLADR-CD11c- * redundant, percent of cell not used in Fig1	B7H3, PDL1, ICOSL, CD86, CD83	CD11c-DR-

Supplementary Table 2. Patient characteristics of the 22 tumors analyzed by flow cytometry per CD3 group

CD3 group	Age	Gender	Site	Stage_UICC	HPV	Diff_Ix	Mit_Ix	PNI	VE	OH	Tobacco	N_status	ECS	Margins
CD3 High	82,8	1	oral cavity	2	unknown	1	unknown	0	0	0	0	0	0	0
CD3 High	46,1	1	oral cavity	3	0	2	unknown	0	1	0	1	1	0	0
CD3 High	90,5	0	oral cavity	4	unknown	1	3	unknown	0	0	1	1	1	0
CD3 High	58,2	0	oral cavity	4	1	2	unknown	1	1	1	1	1	1	2
CD3 High	82,9	0	oral cavity	4	unknown	2	3	0	1	0	0	1	1	0
CD3 High	74,3	1	oropharynx	1	1	2	3	0	0	0	0	0	0	1
CD3 High	54,7	0	oropharynx	3	1	2	3	0	0	0	0	0	0	0
CD3 High	55,3	1	oropharynx	4	0	1	unknown	0	0	1	1	1	1	1
CD3 Int	63,6	0	larynx	4	unknown	1	unknown	1	1	1	1	1	1	0
CD3 Int	63,2	0	oral cavity	2	0	1	3	0	0	0	0	0	0	1
CD3 Int	80,3	1	oral cavity	4	unknown	1	unknown	unknown	unknown	0	1	1	0	unknown
CD3 Int	71,2	1	oral cavity	4	unknown	1	2	0	0	0	0	1	1	0
CD3 Int	67,2	0	oral cavity	4	1	3	3	0	0	1	1	0	0	0
CD3 Int	63,6	0	oropharynx	3	0	3	3	1	0	1	1	0	0	0
CD3 Low	55,3	0	hypopharynx	4	unknown	2	3	1	1	0	1	1	0	1
CD3 Low	63,8	0	larynx	4	0	2	unknown	unknown	unknown	0	1	1	0	unknown
CD3 Low	72,0	0	larynx	4	unknown	2	3	0	0	0	1	0	0	0
CD3 Low	59,3	0	oral cavity	1	unknown	1	unknown	0	0	1	1	0	0	0
CD3 Low	57,8	0	oral cavity	4	0	1	1	0	0	0	0	0	0	0
CD3 Low	50,4	0	oropharynx	1	0	1	2	0	0	1	1	0	0	0
CD3 Low	62,1	0	oropharynx	3	0	1	3	1	1	1	1	0	0	1
CD3 Low	67,6	0	oropharynx	3	unknown	3	3	0	1	1	1	0	0	0

LEGEND

Gender	0=Male, 1=Female
HPV	0=Negative, 1=Positive
Diff_Ix	Differentiation Index: 1=well, 2=moderately, 3=poorly differentiated
Mit_Ix	Mitotic Index: 1=low, 2=intermediate, 3=high
PNI	0=Negative, 1=Positive
VE	0=Negative, 1=Positive
OH	0=Negative, 1=Positive
Tobacco	0=Negative, 1=Positive
N_status	0=Negative, 1=Positive
ECS	0=Negative, 1=Positive
Margins	0=Negative, 1=Close, 2=Positive
Stage_UICC	According to the 7th edition UICC

Supplementary Table 3. In vitro analysis: Percentages of datapoints in each PD-L1/ICOSL category per perturbator

Perturbators	Receptor	**Total number of data points**	**PDL1 Hi / ICOSL Lo**	PDL1 Lo / ICOSL Hi	PDL1 Lo / ICOSL Lo	PDL1 Hi / ICOSL Hi
PAM3 (1ug/ml)	TLR1 :TLR2	2	0%	100%	0%	0%
GMCSF (50ng/ml)	GMCSFR	10	0%	80%	10%	10%
TSLP (50ng/ml)	TSLPR	15	0%	87%	13%	0%
Flu (1X)	TLR7, Cytosolic sensors	16	0%	75%	6%	19%
Med		29	0%	24%	76%	0%
HKCA (MOI1)	Dectin-1	4	0%	0%	100%	0%
HKLM (MOI1)	TLR2	8	13%	38%	50%	0%
Curdlan (10ug/ml)	Dectin-1	8	25%	25%	25%	25%
PAM3 (10ug/ml)	TLR1 :TLR2	8	38%	13%	50%	0%
LPS (100ng/ml)	TLR4	11	45%	27%	18%	9%
PolyIC (50ug/ml)	TLR3	8	50%	38%	0%	13%
Flu (1X) + TSLP	See Flu and TSLP	3	67%	0%	0%	33%
Zymosan (10ug/ml)	TLR2, Dectin-1	8	75%	0%	25%	0%
HKSA (MOI10)	TLR2	4	75%	0%	0%	25%
HKSA (MOI1)	TLR2	6	83%	0%	17%	0%
R848 (1ug/ml)	TLR7/8	13	92%	0%	8%	0%
HKLM (MOI100)	TLR2	1	100%	0%	0%	0%
Total number		154	44	54	46	10

Supplementary Table 4. In vitro analysis: List of measured checkpoints and maturation markers and antibodies list, and corresponding genes.

Marker	Dye	Brand	Clone	Ref	Corresponding genes
4-1BBL	APC	R&D Systems	282220	FAB2295A	TNFSF9
B7H3	FITC	R&D Systems	185504	FAB1027F	CD276
CD100	FITC	BioLegend	A8	328406	SEMA4D
CD11a	PerCP	R&D Systems	CR38	FAB35951C	ITGAL
CD18	PE	BioLegend	TS1/18	302107	ITGB2
CD229/ SLAMF3	APC	R&D Systems	249936	FAB1898A	LY9
CD29	AF700	BioLegend	TS2/16	303020	ITGB1
CD30L	PE	R&D Systems	LQI03	FAB1028P	TNFSF8
CD40	PE-Cy7	BioLegend	5C3	334321	CD40
CD54	BV711	BD	HA58	564078	ICAM1
CD70	FITC	BD	Ki-24	555834	CD70
CD80	BV786	BD	L307.4	564159	CD80
CD83	PerCP/Cy5.5	BioLegend	HB15e	305320	CD83
CD86	BV650	BioLegend	IT2.2	305428	CD86
Galectin 3	AF488	R&D Systems	Polyclonal Goat IgG	IC1154G	LGALS3
HLA-DR	BV711	BioLegend	L243	307644	HLADR
ICAM-2	FITC	BioLegend	CBR-IC2/2	328507	ICAM2
ICAM-3	APC	BioLegend	CBR-IC3/1	330011	ICAM3
ICOSL	APC	R&D Systems	136726	FAB165A	ICOSLG
Jagged 2	APC	BioLegend	MHJ2-523	346906	JAG2
LFA3 / CD58	PE-Cy5	BioLegend	TS2/9	330909	CD58
LIGHT	PE	R&D Systems	115520	FAB664P	TNFSF14
Nectin-2	PE	BioLegend	TX31	337410	NECTIN2
OX40L	R-PE	Ancell	ANC10G1	400-050	TNFSF4
PDL1	PerCP-eFluor e710	eBioscience	MIH18	46-5983-42	CD274
PDL2	BV786	BD	MIH18	563843	PDCD1LG2
PVR	PE	BioLegend	SKII.4	337619	PVR
SLAMF5	FITC	R&D Systems	Polyclonal Goat IgG	FAB1855F	CD84
VISTA	AF700	R&D Systems	730804	FAB71261N	VSIR

Supplementary Table 5. In vitro analysis: List of measured DC Cytokines and Chemokines, and corresponding genes.

Analytes	Methods	Corresponding gene(s)
APRIL	Luminex	TNFSF13
BCA1	Luminex	CXCL13
CCL19	Luminex	CCL19
CXCL11	Luminex	CXCL11
CXCL16	Luminex	CXCL16
CXCL9	Luminex	CXCL9
Eotaxin2	Luminex	CCL24
I309	Luminex	CCL1
IFNa	CBA	IFNA1, IFNA10, IFNA13, IFNA14, IFNA16, IFNA17, IFNA2, IFNA21, IFNA22P, IFNA4, IFNA5, IFNA6, IFNA7, IFNA8
IFNb	Luminex	IFNB1
IL10	CBA	IL10
IL12p40	Luminex	IL12B
IL12p70	CBA	IL12A, IL12B
IL16	Luminex	IL16
IL1a	CBA	IL1A
IL1b	CBA	IL1B
IL1RA	Luminex	IL1RN
IL23	Luminex	IL23A
IL27	Luminex	IL27
IL28a	Luminex	IL28A, IL28B, IFNL2, IFNL3
IL29	Luminex	IL29, IFNL1
IL6	CBA	IL6, IFNL1
IP10	Luminex	CXCL10
MCP1	Luminex	CCL24
MCP2	Luminex	CCL8
MCP4	Luminex	CCL13
MIP1a	Luminex	CCL3
RANTES	Luminex	CCL5
TARC	Luminex	CCL17
TNFa	CBA	TNF
TRAIL	Luminex	TNFSF10
YKL40	Luminex	CHI3L1

Supplementary Table 6. In vitro analysis: List of measured T helper cytokines

Analytes	Technology for measurements
GMCSF	CBA
IFNg	CBA
IL10	CBA
IL13	CBA
IL17A	CBA
IL17F	CBA
IL2	CBA
IL21	Luminex
IL22	Luminex
IL3	CBA
IL31	Luminex
IL4	CBA
IL5	CBA
IL6	CBA
IL9	CBA
TNFa	CBA
TNFb	Luminex

Supplementary Table 7. Bulk RNAseq_DC and MMAC: Characteristics of the 7 patients with tumor and/or blood DC and MMAC were analyzed by RNAseq.

All 7 donors were HNSCC bearing patients		
	Tumor only	n = 4
	Blood only	n = 1
	Tumor and Blood	n = 2

Patients Characteristics		
Age (Mean +/- SD)		63.6 +/- 13.0
SexRatio (%Male)		0,57
T Stage	T3	n=1
	T4	n=6
	N0	n=1
	N+	n=6
Localisation	Oral Cavity	n=5
	Oropharynx HPV+	n=1
	Oropharynx HPV unknown	n=1

Supplementary Table 8. Bulk RNAseq: Tumor Secretory cDC2 Signature.

PVR
NECTIN2
ICAM1
PDCD1LG2
CD40
IL10
IL12A
IL12B
IL1B
TNF
CCL19
CD274
IL6
IL23A
IL27
CCL3
CXCL13
ANKRD33B
AOC1
BCL2L14
BIRC3
CCL17
CCR7
CD83
FSCN1
IL22RA2
LAMP3
MARCKSL1
NCCRP1
RAB9A
SLCO5A1
TBC1D4
TMEM150C
TNFRSF11B
TREML1
WFDC21P

Supplementary Table 9. ScRNAseq_HNSCC: Clusters annotation and cell counts per sample

Cluster	Annotation	Cell Type	Cell Gate	Juxta Pt1	Tumor Pt1	Tumor Pt2	All Tumor Pt1/Pt2	All Samples	% Juxta (nJ/totJ)	%Tumor (nT/totT)	%Tum /% Juxta	% All samples
0	MMAC_PDL1	MMAC	MMAC	327	588	410	998	1325	22,7%	11,0%	0,5	12,62%
1	pDC	pDC	DC/pDC	16	851	384	1235	1251	1,1%	13,6%	12,3	11,91%
2	MMAC_NLRP3	MMAC	MMAC	451	354	137	491	942	31,3%	5,4%	0,2	8,97%
3	CD4_Conv	CD4 T cells	CD3	79	330	456	786	865	5,5%	8,7%	1,6	8,24%
4	CD4_Memory	CD4 T cells	CD3	142	296	207	503	645	9,9%	5,6%	0,6	6,14%
5	CD8_Cytotoxic	CD8 T cells	CD3	57	156	316	472	529	4,0%	5,2%	1,3	5,04%
6	TReg	CD4 T cells	CD3	36	334	105	439	475	2,5%	4,8%	1,9	4,52%
7	cDC2	cDC	DC/pDC	54	192	227	419	473	3,8%	4,6%	1,2	4,50%
8	Plasma cells	B cells	Others	9	178	260	438	447	0,6%	4,8%	7,7	4,26%
9	B cells	B cells	Others	8	81	355	436	444	0,6%	4,8%	8,7	4,23%
10	CD8_Memory	CD8 T cells	CD3	46	149	168	317	363	3,2%	3,5%	1,1	3,46%
11	Neutrophils	Neutrophils	Others	9	20	310	330	339	0,6%	3,6%	5,8	3,23%
12	MMAC_C1Q	MMAC	MMAC	50	194	89	283	333	3,5%	3,1%	0,9	3,17%
13	TReg_PD1	CD4 T cells	CD3	34	229	65	294	328	2,4%	3,2%	1,4	3,12%
14	Mast cells	Mast cells	Others	18	186	122	308	326	1,3%	3,4%	2,7	3,10%
15	CD8_Exhausted	CD8 T cells	CD3	40	121	146	267	307	2,8%	2,9%	1,1	2,92%
16	pDC_TCL1A	pDC	DC/pDC	1	119	112	231	232	0,1%	2,5%	36,7	2,21%
17	NK cells	NK cells	Others	20	48	134	182	202	1,4%	2,0%	1,4	1,92%
18	Cancer cells	Cancer cells	CD45neg	1	41	156	197	198	0,1%	2,2%	31,3	1,89%
19	Erythrocytes	Red Blood cells	CD45neg	7	139	7	146	153	0,5%	1,6%	3,3	1,46%
20	Mature migratory cDC	cDC	DC/pDC	7	52	52	104	111	0,5%	1,1%	2,4	1,06%
21	Tcell_Cycling	T cells	CD3	12	73	2	75	87	0,8%	0,8%	1	0,83%
22	Fibroblasts	Fibroblasts	CD45neg	10	8	55	63	73	0,7%	0,7%	1	0,70%
23	cDC1	cDC	DC/pDC	6	21	28	49	55	0,4%	0,5%	1,3	0,52%
TOTAL				1440	4760	4303	9063	10503	100,00%	100,00%	1	100,00%

Supplementary Table 10. Mature migratory DC (mmDC) Signature.

This 29-gene signature was obtained used genes in common between cluster 20 signature in Supplementary Data 10 (mmDC in our ScRNA HNSCC data) and cluster 4 signature in Supplementary Data 20 (mmDC in the merged ScRNA datasets.)

FSCN1
LAMP3
TXN
GPR157
CD274
BIRC3
CSF2RA
CCR7
CD40
NUB1
EBI3
POGLUT1
CCL19
CCL22
MARCKS
MARCKSL1
TNFAIP2
RAB9A
MGLL
ANXA6
DUSP5
ID2
LAD1
FAM49A
CXCL9
HSPB1
PLA2G16
KIF2A
CSTA
