# Supplementary material for: PD-L1 and ICOSL discriminate human Secretory and Helper dendritic cells in cancer, allergy and autoimmunity
Source: Nat Commun. 2022 Apr 13;13:1983. doi: 10.1038/s41467-022-29516-w (PMC9008048; doi:10.1038/s41467-022-29516-w)
Supplement: Supplementary file 3 — Description of Additional Supplementary Files [file 41467_2022_29516_MOESM3_ESM.pdf]

Description of Additional Supplementary Files

File Name: Supplementary Data 1
Description: Spearman correlation matrix of the non-redundant parameters measured by Flow Cytometry presented in Figure 1

File Name: Supplementary Data 2
Description: Bulk RNAseq_cDC2: Differentially expressed genes (DEG) between Blood and Tumor cDC2 at FDR 0.1

File Name: Supplementary Data 3
Description: Bulk RNAseq_Public data: List of genes associated with the Venn diagram comparing DEG between pRNA and GMCSF at FDR 0.1

File Name: Supplementary Data 4
Description: List of 148 genes used for supervised analysis of checkpoints and maturation markers

File Name: Supplementary Data 5
Description: List of 117 genes used for supervised analysis of cytokines

File Name: Supplementary Data 6
Description: List of 52 genes used for supervised analysis of chemokines

File Name: Supplementary Data 7
Description: List of 100 genes used for supervised analysis of the NFkB pathway

File Name: Supplementary Data 8
Description: Bulk RNAseq_DC and MMAC: Tumor DC and MMAC subsets signatures

File Name: Supplementary Data 9
Description: ScRNAseq_HNSCC: DEG per cluster

File Name: Supplementary Data 10
Description: ScRNAseq_HNSCC: DEG per cluster_filtered L2FC>1 and padj <0.05

File Name: Supplementary Data 11
Description: Antibodies and Panels list for samples phenotyping and/or sorting

File Name: Supplementary Data 12
Description: List of 119 genes used for supervised analysis of Innate Receptors

File Name: Supplementary Data 13
Description: ScRNAseq_HNSCC: ICELLNET IN communication scores

File Name: Supplementary Data 14
Description: ScRNAseq_HNSCC: ICELLNET OUT communication scores

File Name: Supplementary Data 15
Description: List of datasets merged for DC heterogeneity and maturation analysis

File Name: Supplementary Data 16
Description: ScRNAseq_Cillo et al._DEG per cluster

File Name: Supplementary Data 17
Description: ScRNAseq_Luminal Breast Cancer_DEG per cluster

File Name: Supplementary Data 18
Description: ScRNAseq_He et al._DEG per cluster

File Name: Supplementary Data 19
Description: ScRNAseq_Merged DC Datasets: DEG per cluster

File Name: Supplementary Data 20
Description: ScRNAseq_Merged DC Datasets: DEG per cluster_filtered L2FC>1 and padj <0.05

File Name: Supplementary Data 21
Description: ScRNAseq_Merged DC Datasets: Number and percentage of cells per cluster per sample

File Name: Supplementary Data 22
Description: ScRNAseq_Merged DC Datasets: ANOVA of Tumor Secretory DC, pRNA Secretory DC and GM-CSF Helper DC signatures
