# Supplementary material for: PD-L1 and ICOSL discriminate human Secretory and Helper dendritic cells in cancer, allergy and autoimmunity
Source: Nat Commun. 2022 Apr 13;13:1983. doi: 10.1038/s41467-022-29516-w (PMC9008048; doi:10.1038/s41467-022-29516-w)
Supplement: Supplementary file 26 — Reporting Summary [file 41467_2022_29516_MOESM26_ESM.pdf]

nature research

Corresponding author(s): Vassili Soumelis, Caroline Hoffmann

Last updated by author(s): Jan, 4th, 2022

# Reporting Summary

Nature Research wishes to improve the reproducibility of the work that we publish. This form provides structure for consistency and transparency in reporting. For further information on Nature Research policies, see Authors & Referees and the Editorial Policy Checklist.

## Statistics

For all statistical analyses, confirm that the following items are present in the figure legend, table legend, main text, or Methods section.

| n/a | Confirmed | |
|---|---|---|
| ☐ | ☒ | The exact sample size (*n*) for each experimental group/condition, given as a discrete number and unit of measurement |
| ☐ | ☒ | A statement on whether measurements were taken from distinct samples or whether the same sample was measured repeatedly |
| ☐ | ☒ | The statistical test(s) used AND whether they are one- or two-sided<br>*Only common tests should be described solely by name; describe more complex techniques in the Methods section.* |
| ☐ | ☒ | A description of all covariates tested |
| ☐ | ☒ | A description of any assumptions or corrections, such as tests of normality and adjustment for multiple comparisons |
| ☐ | ☒ | A full description of the statistical parameters including central tendency (e.g. means) or other basic estimates (e.g. regression coefficient) AND variation (e.g. standard deviation) or associated estimates of uncertainty (e.g. confidence intervals) |
| ☐ | ☒ | For null hypothesis testing, the test statistic (e.g. *F*, *t*, *r*) with confidence intervals, effect sizes, degrees of freedom and *P* value noted<br>*Give P values as exact values whenever suitable.* |
| ☒ | ☐ | For Bayesian analysis, information on the choice of priors and Markov chain Monte Carlo settings |
| ☒ | ☐ | For hierarchical and complex designs, identification of the appropriate level for tests and full reporting of outcomes |
| ☐ | ☒ | Estimates of effect sizes (e.g. Cohen's *d*, Pearson's *r*), indicating how they were calculated |

*Our web collection on statistics for biologists contains articles on many of the points above.*

## Software and code

Policy information about availability of computer code

| | |
|---|---|
| Data collection | RNAseq reads were mapped to the human genome reference (hg19/GRCh37) using Tophat2 software version 2.0.14. Gene expression values were quantified as read counts using HTSeq-count version 0.6.1. For Single-cell transcriptomic data, FASTQ files were processed using the Cell Ranger pipeline (10X Genomics; V3 for HNSCC samples and V2 for Breast cancer sample), and reads were aligned to the human genome hg38. Computer code is available on demand by contacting the corresponding author. The count matrix and associated metadata of the merged cDC meta-dataset related to Figure 8 has been deposited in the NCBI under the identification number GSE170673. |
| Data analysis | FlowJo V10; Graphpad Prism version 8; Qlucore version 3.5; R software version 3.5.3, 3.6.1, and 3.6.3; DESeq2 R package version 1.26.0; Seurat version 3.2.2; Dropletutils R package version 1.6.1; CITE-seq Count version 1.4.3; ICELLNET (https://github.com/soumelis-lab/ICELLNET); Monocle 3 version 0.2.2. |

For manuscripts utilizing custom algorithms or software that are central to the research but not yet described in published literature, software must be made available to editors/reviewers. We strongly encourage code deposition in a community repository (e.g. GitHub). See the Nature Research guidelines for submitting code & software for further information.

## Data

Policy information about availability of data

All manuscripts must include a data availability statement. This statement should provide the following information, where applicable:

- Accession codes, unique identifiers, or web links for publicly available datasets
- A list of figures that have associated raw data
- A description of any restrictions on data availability

The source data file contains the raw data for Figures 3C; S5B; S6A-B. The RNAseq and ScRNAseq data generated in this study have been deposited in the NCBI under the identification numbers GSE169381 and GSE170673. Reads were aligned to the human genome hg19 (bulk RNAseq) and hg38 (Single-cell RNAseq). Other datasets were used in this study: Mathan et al. (GSE89442); Cillo et al. (GSE139324); Zillionis et al. (GSE127465); He et al. (GSE147424); Arazi et al. (https://doi.org/10.1038/s41590-019-0398-x); Riaz et al. (https://github.com/riazn/bms038_analysis/tree/master/data); Gide et al. (https://github.com/miabioinformatics/

Gide_CancerCell2019); TCGA (https://portal.gdc.cancer.gov); Metabric data (Curtis et al., Nature. 2012 Apr 18;486(7403):346–52); DC-T database (source of Figures 2; 3A-B; S4 A-D) is from Grandclaudon et al., Cell. 2019 Oct 3;179(2):432-447.e21 (see Table S2 in this reference). The Flow cytometry data (Figure 1; S2B; S3A-B) are available under restricted access for other ongoing projects: access can be obtained by contacting the corresponding author.

# Field-specific reporting

Please select the one below that is the best fit for your research. If you are not sure, read the appropriate sections before making your selection.

☒ Life sciences ☐ Behavioural & social sciences ☐ Ecological, evolutionary & environmental sciences

For a reference copy of the document with all sections, see nature.com/documents/nr-reporting-summary-flat.pdf

# Life sciences study design

All studies must disclose on these points even when the disclosure is negative.

**Sample size**

For the in vitro analyses, the large number of individual experiments performed (154 and 130) ensure sufficient sample size, and no prior sample size calculation was performed. For the human sample analysis, sample size was determined based on availability and resources, nd no prior sample size calculation was performed.

**Data exclusions**

For survival analyses, two patients with metastatic head and neck cancer in the TCGA database for prognosis analysis, because their disease stage and survival outcomes was not comparable to the other 500 patients. Patients with incomplete survival data from the METABRIC database were also excluded.

**Replication**

A large number of experiments (154 and 130) were performed for the in vitro analyses to ensure reproducibility of the results. No technical replicates were made. Due to the rareness and limited size of the human-derived samples, we could not perform replicates, but favored analysing the maximum number of samples available.

**Randomization**

Randomization was not relevant to this study because there was no experimental groups.

**Blinding**

Blinding was not relevant to this study,because there was no experimental groups.

# Reporting for specific materials, systems and methods

We require information from authors about some types of materials, experimental systems and methods used in many studies. Here, indicate whether each material, system or method listed is relevant to your study. If you are not sure if a list item applies to your research, read the appropriate section before selecting a response.

## Materials & experimental systems

| n/a | Involved in the study | |
|---|---|---|
| ☐ | ☒ | Antibodies |
| ☒ | ☐ | Eukaryotic cell lines |
| ☒ | ☐ | Palaeontology |
| ☒ | ☐ | Animals and other organisms |
| ☐ | ☒ | Human research participants |
| ☐ | ☒ | Clinical data |

## Methods

| n/a | Involved in the study | |
|---|---|---|
| ☒ | ☐ | ChIP-seq |
| ☐ | ☒ | Flow cytometry |
| ☒ | ☐ | MRI-based neuroimaging |

## Antibodies

**Antibodies used**

CD14-FITC (BD; clone:M5E2; ref555397; lot:3163943 and 6263655), PDL1-PerCP eFluor 710 (Ebiosciences; clone:MIH1; ref46-5983-42; lot:E151400-105), ICOSL-APC (R&D; clone:136726; refFAB165A; lot:AALD0216031), CD3-Alexa-700 (BD; clone:UCHT1; ref557943; lot:6043535 and 7052530), CD19-Alexa-700 (BD; clone:HIB19; ref557921; lot:5226800 and 7045883), CD56-Alexa-700 (BD; clone:B159; ref557919; lot:6126945), CD45-APC-Cy7 (BD; clone:2D1; ref557833; lot:5271696), CD83-BV605 (BD; clone:HB15e; ref740420; lot:6155784), CD123-BV650 (BD; clone:7G3; ref563405; lot:6095619), B7H3-PE (Biolegend; clone:MIH42; ref351004; lot:B191890), CD11c-PECF594 (BD; clone:B-ly6; ref562393; lot:4127693), CD1c/BDCA1-PC7 (Biolegend; clone:L161; ref331516; lot:B189320), HLA-DR-BUV395 (BD; clone:G 46-6; ref564040; lot:7030621), CD86-BUV737 (BD; clone:2331 (FUN-1); ref564428; lot:6123528), CD15-BV785 (Biolegend; clone:W6D3; ref323043; lot:B246887), CD14-Qdot 605 (Life technology; clone:TuK4; refQ10013; lot:1719149), HLA-DR-BV711 (Biolegend; clone:L243; ref307643; lot:B221994), BDCA-3-APC (Miltenyi; clone:AD5-14H12; ref130-113-876; lot:B155625), CD1a-PC5 (BD ; clone:HI149; ref555808; lot:35647), CD11c-PC7 (Biolegend; clone:Bu15; ref337216; lot:B224435), "BDCA-1-PE (Biolegend; clone:L161; ref331506; lot:B155796), CD123-Fluo 650NC (eBiosciences; clone:6H6; ref95-1239-42; lot:E10545-1633), Langerin-FITC (Miltenyi; clone:MB22-9F5; ref130-098-349; lot:5131121265), CD45-BV570 (Biolegend; clone:HI30; ref304033; lot:B156208), CD14-FITC (Biolegend; clone:10.1; ref555527; lot:2359722), HLA-DR-APC-eFluor780 (Ebiosciences; clone:LN3; ref47-9956-42; lot:E11367-1633), CD11c-PECy5 (BD; clone:B-ly6; ref551077; lot:3192777), FceR1-APC (Ebiosciences; clone:AER-37; ref17-5899-42; lot:E12842-104), TCRgd-FITC (BD; clone:11F2; ref347903; lot:5327683), TCRVa7.2 -PerCP 5.5 (Biolegend; clone:3C10; ref351710; lot:B189338), ICOS -APC (Ebiosciences; clone:ISA-3; ref17-9948-42; lot:E12287-1639), CD3 -Alexa700 (Biolegend; clone:UCHT1; ref300424;

lot:B216765), CD57-APC Vio770 (Miltenyi; clone:TB03; ref130-104-197; lot:5160407209), TCR Va24 -BV510 (Biolegend; clone:6B11; ref342918; lot:B209298), CD27 -BV605 (Biolegend; clone:O323; ref302830; lot:B202175), CD127 -BV650 (Biolegend; clone:A019D5; ref351326; lot:B191728), PD1 -BV711 (Biolegend; clone:EH12.2H7; ref329928; lot:B196373), CD161-BV785 (Biolegend; clone:HP-3G10; ref339930; lot:B215693), CD25-PE (BD; clone:M-A251; ref555432; lot:6040885), CD4 -PE TX (Invitrogen; clone:S3.5; refMHCD0417; lot:1576408A), CD8b-PC5 (Coulter; clone:2ST8.5H7; ref6607109; lot:7623012), CD45RA-PC7 (Ebiosciences; clone:HI100; ref25-0458-42; lot:E09825-1638), CD123-PerCPCy5.5 (BD; clone:7G3; ref560904; lot:7208636), CD19-APC (Biolegend; clone:HIB19; ref302211; lot:B245676), CD56-APC (Biolegend; clone:HCD56; ref318309; lot:B235382), CD235a-BV421 (BD; clone:GA-R2 (HIR2); ref562938; lot:8201995), HLA-DR-BV711 (Biolegend; clone:L243; ref307644; lot:B221994), CD3-APC (BD; clone:UCHT1; ref555335; lot:7200698), CD1c/BDCA1-PE (Biolegend; clone:L161; ref331506; lot:B155796), TOTALSEQ-A ANTIBODY ANTI-CD4- (Biolegend; clone:RPA-T4; ref300563; lot:B268766), TOTALSEQ-A ANTIBODY ANTI-CD8- (Biolegend; clone:SK1; ref344751; lot:B260214), TOTALSEQ-A ANTIBODY ANTI-TCRgd- (Biolegend; clone:B1; ref331229; lot:B260353), TOTALSEQ-A ANTIBODY ANTI-CD25- (Biolegend; clone:BC96; ref302643; lot:B269310), TOTALSEQ-A ANTIBODY ANTI-CD103- (Biolegend; clone:Ber-ACT8; ref350231; lot:B263617), TOTALSEQ-A ANTIBODY ANTI-CD27- (Biolegend; clone:O323; ref302847; lot:B263620), TOTALSEQ-A ANTIBODY ANTI-CD15- (Biolegend; clone:W6D3; ref323046; lot:B261038), TOTALSEQ-A ANTIBODY ANTI-CD1c- (Biolegend; clone:L161; ref331539; lot:B259973), TOTALSEQ-A ANTIBODY ANTI-PDL1/CD274- (Biolegend; clone:29E2A3; ref329743; lot:B263612), TOTALSEQ-A ANTIBODY ANTI-ICOSL/CD275- (Biolegend; clone:2D3; ref309413; lot:B263787), CD3-FITC (BD; clone:HIT3a; ref:555339; lot:8333818), CD14-FITC (Miltenyi; clone:TÜK4; ref:130-080-701; lot:5191023186), CD16-FITC (BD; clone:NKP15; ref:335035; lot:8089995), CD19-FITC (Miltenyi; clone:LT19; ref:130-091-328; lot:5191022444), CD20-FITC (BD; clone:2H7; ref:555622; lot:7313759), CD56-FITC (Biolegend; clone:HCD56; ref:318303; lot:B279065), CD11c-PECy7 (Biolegend; clone:Bu15; ref:337216; lot:B289308), CD1c/BDCA1-eFluor 710 (Thermofisher; clone:L161; ref:46-0015-52; lot:4336341), CD123-BV650 (Biolegend; clone:7G3; ref:306020; lot:B207937), CD141-PE (Miltenyi; clone:REA674; ref:130-110-258; lot:5180706304). Dilutions are available in the table "Materials".

Validation

All antibodies (except Totalseq-A) were previously titrated using peripheral blood mononuclear cells from healthy donors or in digested primary tumor samples, or in DC-enriched fraction of PBMC for DC-specific markers (CD1c; CD141).
Totalseq-A antibodies were used as per 10X Genomics protocole: 1µg for 1-2 million cells in 100µl of staining buffer (PBS 1X (Gibco) supplemented with EDTA 2 mM (Gibco) and 1% de-complemented human serum (BioWest)).

## Human research participants

Policy information about studies involving human research participants

Population characteristics

Inclusion criteria were patients with previously untreated head and neck squamous cell carcinoma, aged over 18. Detailed patients characteristics are available in Table 3 and Table 8. Exclusion criteria were concurrent

Recruitment

Patients meeting inclusion criteria were prospectively recruited by the physicians after signing an informed consent. There was no specific patient selection.

Ethics oversight

This study was approved by the Internal Review Board and Clinical Research Committee of the Institut Curie.

Note that full information on the approval of the study protocol must also be provided in the manuscript.

## Clinical data

Policy information about clinical studies
All manuscripts should comply with the ICMJE guidelines for publication of clinical research and a completed CONSORT checklist must be included with all submissions.

Clinical trial registration

Fourteen of the 22 patients of the flow cytometry cohort were included in the observational clinical trial SCANDARE NCT03017573

Study protocol

Full study protocol can be accessed by contacting maud.kamal@curie.fr or christophe.letourneau@curie.fr

Data collection

Recruitment was performed at the Institut Curie Hospital and data was collected at the Institut Curie Research Center in the INSERM U932 research unit, between January 2014 and December 2018.

Outcomes

Outcomes was not relevant to this study because there it was observational

## Flow Cytometry

### Plots

Confirm that:

☒ The axis labels state the marker and fluorochrome used (e.g. CD4-FITC).

☒ The axis scales are clearly visible. Include numbers along axes only for bottom left plot of group (a 'group' is an analysis of identical markers).

☒ All plots are contour plots with outliers or pseudocolor plots.

☒ A numerical value for number of cells or percentage (with statistics) is provided.

## Methodology

Sample preparation

Tumor tissues were mechanically and enzymatically digested in CO2-independent medium (Gibco) containing 5% FBS (HyClone). Enzymatic digestion consisted of three rounds of 15 min of incubation with agitation at 37 °C, separated by pipetting, with 2 mg/ml collagenase I (C0130, Sigma), 2 mg/ml hyaluronidase (H3506, Sigma) and 25 µg/ml DNAse (Roche). The samples were filtered on a 40-µm cell strainer (Fischer Scientific) and were diluted in PBS 1X (Gibco) supplemented with EDTA 2 mM (Gibco) and 1% de-complemented human serum (BioWest). After centrifugation, cells were suspended in the same medium and were counted by trypan blue before being assessed by flow cytometry or sorted. PBMC were isolated from blood samples using FICOLL (GE Healthcare) gradient centrifugation. Single-cell suspensions from digested tumor and from blood were stained with antibodies (Table "Materials") for 15 min at 4°C. After washing step, cells were analyzed or sorted directly, immediately after having added DAPI (Miltenyi Biotec) for dead cells exclusion.

Instrument

BD LSR Fortessa and BD FACS Aria III

Software

FlowJo10.4 for anlayses; FACS DIVA 6 for sorting

Cell population abundance

0.2 to 6.1% among CD45+ tumor single cell suspension, depending on the APC subset.

Gating strategy

For all flow cytometry and flow sorting analyses, we performed a standard elimination of debris and doublets by using FSC-SCC parameters. Then, dead cells were excluded (positive for DAPI). In the flow cytometry myeloid cell panel in Fig 1, in the flow cytometry CD141 panel in Fig S2B in the flow cytometry CD15 panel in Fig S2C, and in the flow sorting for the RNAseq cohort in Fig 4, the initial gates were as follows: we selected CD45+ cells, then excluded T cells, B cells, and NK cells based on CD3, CD19, and CD56, respectively, in a "lineage" (Lin) panel. This initial gating strategy is shown in Fig S2A. Then, cells were analyzed by their expression of CD11c and HLA-DR. The double positive CD11c+HLADR+ population was separated into four populations based on CD14 and BDCA1, and included the monocytes and macrophages (MMAC (CD14+CD1c-), the CD14+DC (CD1c+CD14+), the cDC2 (CD1c+CD14-) and the double negative population "DN DC/MMAC" (CD1c-CD14-), as shown in Fig1B. Additionally, for the flow cytometry myeloid cell panel in Fig 1, we measured CD11c-HLA-DR+ cells and among them the plasmacytoid DC (pDC) that were gated as CD123+; we measured among Lin-HLA-DR- cells the CD11c+ and the CD11c-. Finally, we measured PD-L1, ICOSL, CD83, CD86 and B7-H3 expression in MMAC, CD14+DC, cDC2, DN DC/MMAC, pDC, CD11c+HLA-DR-, CD11c-HLADR- cells. Additionally, in the flow cytometry CD141 panel in Fig S2B left, we measured the CD141 high cells from the DN DC/MMAC gate; we also quantified CD1a+ and Langerin+ cells from the cDC2 gate, but this data was not used in the present study. Additionally, for the flow cytometry CD15 panel in Figure S2C: we gated Lin-HLA-DR- cells and measured CD15 expression in the CD11c+ and CD11- cell populations. Additionally, for the flow sorting for the RNAseq cohort in Fig 4, we gated the CD11c-HLA-DR+ cells and among them sorted the plasmacytoid DC (pDC) that were gated as CD123+; we sorted the MMAC (CD14+CD1c-), the CD14+DC (CD1c+CD14+), and the cDC2 (CD1c+CD14-) as described above and displayed in Fig S7. For the flow cytometry T cell panel in Fig 1 the full gating strategy is displayed in Fig S1. The sorting of pure cDC2 is shown in Fig S5A: we excluded debris and doublets by FSC, SSC gating; dead cells by Live/Dead Aqua gating, then we selected the CD3-CD14-CD16-CD19-CD20-CD56- cells, then the CD11c+CD123- and finally sorted the CD1c+CD141-. For the ScRNAseq HNSCC samples, the gating strategy is displayed in Fig S10. We excluded debris and doublets by FSC, SSC gating; dead cells by DAPI gating; red blood cells by CD235a staining (Pt2 only). All remaining cells were sorted in one of the 5 final population groups. Group 1 included CD45+CD3+ T cells; Group 2 included non-immune CD45- cells; Group 3 included MMAC and CD14+DC gated as CD45+CD3-CD19-CD56-CD11c+HLA-DR+CD14+; Group 4 included cDC1 and cDC2 gated as CD45+CD3-CD19-CD56-CD11c+HLA-DR+CD14- and was merged with pDC gated as CD45+CD3-CD19-CD56-CD11c-HLA-DR+CD123+; Group 5 included all remaining cells: CD45+CD3-CD19+ and/or CD56+, CD45+CD3-CD19-CD56-CD11c-HLA-DR+CD123-, and CD45+CD3-CD19-CD56-HLA-DR-. For the ScRNAseq luminal breast cancer sample, the gating strategy for DC included the following steps: (i) exclusion of debris and doublets by FSC, SSC gating; (ii) exclusion of dead cells by DAPI gating; (iii) selection of cells positive in CD45 BV570; (iv) exclusion of T cells, identified as CD3+ APC and CD19- Alexa700 and CD56- Alexa700; (v) exclusion of CD19+ Alexa700 and CD56+ Alexa700 cells; (vi) selection of CD11c- PECy5 cells, among which cells CD123+ BV650 and HLA-DR+ eFluor760 double positive were sorted as pDC; besides, selection of CD11c+ PECy5 and HLA-DR+ eFluor760 double positive cells, among which cells were classified according to CD1C PE and CD14 FITC: MMAC defined as CD1C- and CD14+ were excluded and all the other cells were sorted as cDC. Eventually, the DC sample used for ScRNA-seq included the pDC and the cDC. pDC were excluded bioinformatically before the analysis in Fig 8.

☒ Tick this box to confirm that a figure exemplifying the gating strategy is provided in the Supplementary Information.
